# The Next Generation Fecal Microbiota Transplantation: To Transplant Bacteria or Virome

**DOI:** 10.1002/advs.202301097

**Published:** 2023-11-01

**Authors:** You Yu, Weihong Wang, Faming Zhang

**Affiliations:** ^1^ Department of Microbiota Medicine & Medical Center for Digestive Diseases The Second Affiliated Hospital of Nanjing Medical University Nanjing 210011 China; ^2^ Key Lab of Holistic Integrative Enterology Nanjing Medical University Nanjing 210011 China; ^3^ Department of Microbiota Medicine Sir Run Run Hospital Nanjing Medical University Nanjing 211166 China

**Keywords:** bacteriophages, fecal microbiota transplants, fecal virome transplantations, spores, transendoscopic enteral tubing, viromes, washed microbiota transplantations

## Abstract

Fecal microbiota transplantation (FMT) has emerged as a promising therapeutic approach for dysbiosis‐related diseases. However, the clinical practice of crude fecal transplants presents limitations in terms of acceptability and reproductivity. Consequently, two alternative solutions to FMT are developed: transplanting bacteria communities or virome. Advanced methods for transplanting bacteria mainly include washed microbiota transplantation and bacteria spores treatment. Transplanting the virome is also explored, with the development of fecal virome transplantation, which involves filtering the virome from feces. These approaches provide more palatable options for patients and healthcare providers while minimizing research heterogeneity. In general, the evolution of the next generation of FMT in global trends is fecal microbiota components transplantation which mainly focuses on transplanting bacteria or virome.

## Graphical abstract

The use of human feces for treating diseases has a long history, but its rough form is not accepted in modern times. Therefore, new technologies for microbiota transplantation are developed, such as washed microbiota transplantation, bacteria spores treatment, and fecal virome transplantation. This work aims to analyze and showcase the new generation of fecal microbiota transplantation.

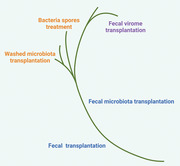

## 1 Introduction

The human gut microbiome is a complex community consisting of bacteria, archaea, viruses, fungi, eukaryotic parasites, protozoa, and their genomes.^[^ ^1^, ^2^ ^]^ Researchers are increasingly interested in treatments targeting the gut microbiome that plays a pivotal role in human health.^[^ ^3^ ^]^ Fecal microbiota transplantation (FMT) is a promising treatment for diseases related to gut dysbiosis,^[^ ^4^, ^5^ ^]^ as it can help to reconstruct the composition and function of imbalanced gut microbiota by transferring fecal preparations from healthy donors.^[^ ^6^ ^]^ A recent systematic review revealed that FMT has been reported to treat 85 specific diseases in clinical settings globally from 2011 to 2021.^[^ ^7^ ^]^ In particular, FMT is highly effective in treating recurrent *Clostridiodes difficile* infection (CDI), with a cure rate of ≈90%.^[^ ^8^ ^]^

Each gram of human feces contains ≈10^11^ bacterial cells, 10^8^‐10^9^ virus‐like particles (most are bacteriophages), ≈10^7^ colonocytes, ≈10^8^ archaea, ≈10^6^ fungi, protists, and metabolites.^[^ ^9^, ^10^, ^11^ ^]^ The transferred microbiota and metabolites can partly explain the efficacy of FMT. For example, metabolites derived from the Firmicutes phylum, specifically short‐chain fatty acids, and secondary bile acids, play multiple beneficial roles in maintaining host homeostasis. These roles include fortifying the gut barrier and mitigating inflammation.^[^ ^12^, ^13^ ^]^ However, varied FMT methods involving varying doses and delivery routes^[^ ^14^ ^]^ have caused different clinical responses among different research teams.^[^ ^15^ ^]^ This heterogeneity presents challenges to research reproducibility. In a parallel progression akin to drug development's evolution from natural formulations to more precise components, FMT is also expected to follow a similar trajectory. Presently, the focus is shifting toward the transplantation of more precise and efficacious microbial components. Both bacteria and the virome, as predominant constituents of the gut microbiota, are believed to play pivotal roles in the effectiveness of FMT.^[^ ^16^, ^17^ ^]^ In this context, researchers and clinicians have turned to methods that involve transplanting bacteria or virome.^[^ ^18^, ^19^, ^20^, ^21^ ^]^

**Figure 1:**
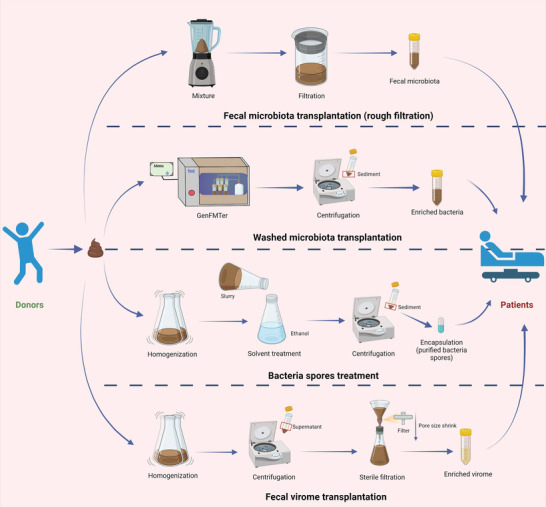
The conceptual graphs of preparation methodology for fecal transplants. The preparation methods for fecal transplants: fecal microbiota transplantation (rough filtration), washed microbiota transplantation, bacteria spores treatment, and fecal virome transplantation. Additional details can be found in Table 1.

Given the abundance of bacteria in the gut microbiome, bacteria have been the focus of most FMT‐related studies.^[^ ^22^ ^]^ Transplanting bacteria has emerged as a promising strategy for refining FMT with improved methodology.^[^ ^23^, ^24^, ^25^ ^]^ The automatic washing process which involves delivering considerations, was coined as washed microbiota transplantation (WMT)^[^ ^26^ ^]^ and released as consensus by the FMT‐standardization Study Group in 2019.^[^ ^27^ ^]^ The primary objective of WMT is to transplant the enriched bacteria using a washed fecal microbiota preparation involving the elimination of bacterial fragments, metabolites, soluble molecules, proteins, and viruses to the greatest extent possible, while also ensuring the removal of all fungi and parasite eggs (**Figure** **1**).^[^ ^26^ ^]^ Another method for transplanting bacteria is bacteria spores treatment, which enriches bacteria spores through a preparation that incorporates chemical and physical methods (Figure 1).^[^ ^25^ ^]^ In recent years, the importance of the virome in gut microbiota has also been increasingly recognized.^[^ ^28^, ^29^ ^]^ However, the methods of WMT and bacteria spores treatment are not actually aimed at transplanting the viral community. When simultaneously considering the transplantation of both bacterial and viral communities, it would involve going back to transplanting fecal matter in its original form, which is unacceptable to doctors and patients.^[^ ^30^, ^31^, ^32^ ^]^ Separating the virome for study purposes could offer a solution, enabling the investigation of the mechanism and potential applications of the virome. The gut virome is mainly composed of prokaryotic viruses,^[^ ^33^ ^]^ with bacteriophages being the dominant type. The total number of gut bacteriophages is estimated to be ≈10^15^.^[^ ^34^ ^]^ Recently, there has been growing interest in fecal virome transplantation (FVT) or fecal filtrate transplantation (FFT), which involve transplanting virome (containing viruses, metabolites, and cellular debris, but not intact bacterial cells) from donor feces to patients, to eliminate adverse events (AE) caused by bacteria (Figure 1).^[^ ^19^, ^35^ ^]^ In 2017, Ott et al. reported clinical success in treating CDI by FFT,^[^ ^19^ ^]^ which has spurred further research into the potential benefits of the virome.^[^ ^22^ ^]^ Of note, to facilitate understanding, we use the term FVT to collectively refer to methods of transplanting fecal virome, which shares similar concepts and methods with FFT.

In the current stage, as the mechanism is partially defined, the next generation of FMT could fulfill the following criteria: uncultured microbes derived from healthy human donors, relative specificity in compositions, higher safety through clinical trials, and gastrointestinal tract as the delivery route. These criteria aim to retain both known and unknown beneficial components in fecal transplants while reducing study heterogeneity through more precise compositions and refined methodologies. In general, the next generation of FMT is progressing towards the direction of transplanting specific microbiota components, namely microbiota components transplantation, mainly targeting bacteria and virome. In this review, the methods involved in transplanting bacteria and viromes meet these criteria. Although their primary components differ, their mechanisms are interconnected and deserve further exploration. It is worth noting that certain promising products with well‐defined and reproducible components, including formula (or cocktail) bacteria,^[^ ^36^ ^]^ bacteriophage cocktail treatment,^[^ ^37^ ^]^ and bacteriophage consortia treatment,^[^ ^38^ ^]^ are excluded in this review.

In this review, we discussed milestones of FMT in the medical history, and the safety and regulation concerns associated with FMT. We also displayed the challenges facing FMT and emphasized the importance of improving its methodology. Finally, we discussed cutting‐edge research on transplanting bacteria or virome, including their methodology, applications, safety, potential mechanism, research techniques, and prospects. Our goal was to offer guidance for the future development of microbiota‐based therapies and enrich the scientific explanations of microbiota medicine^[^ ^39^ ^]^ as a branch discipline of clinical medicine.

## 2 The Development of FMT

### 2.1 The Milestones of FMT in the Medical History

The first milestone of FMT in medical history should be the ancient record in traditional Chinese medicine. The use of FMT for treating human diseases can be traced back to at least the 4^th^ century, as documented in the Zhou Hou Bei Ji Fang, a Chinese handbook of emergency medicine.^[^ ^5^ ^]^ According to this ancient medical history, traditional Chinese medicine doctors used oral human fecal suspension to treat patients with food poisoning or severe diarrhea. This historical account meets several criteria that confirm the use of FMT, including the use of human fecal matter as the source material, administration via the digestive tract, efficacy mediated by the microbiota present in fresh fecal water or fermented fecal matter, and clear descriptions of prescriptions, methods, indications, and efficacy in ancient literature.^[^ ^5^ ^]^

In 1958, FMT through enema was first reported to treat pseudomembranous enterocolitis.^[^ ^40^ ^]^ In 1983, Schwan et al. reported the first case of recurrent CDI successfully treated with homologous FMT.^[^ ^41^ ^]^ In 2013, FMT was first time recommended for the treatment of recurrent CDI by the guideline led by Surawicz.^[^ ^42^ ^]^ This is a milestone for FMT from folk remedies to recommendations by professional teams. In modern medicine, the range of diseases being investigated for treatment with FMT has significantly expanded, but CDI remains the only well‐established indication.^[^ ^4^, ^5^ ^]^ The data as of August 2023 shows that the disease with the highest number of registered clinical trials for FMT treatment on the clinicaltrials.gov website is IBD. From 2011 to 2021, a total of 2,400 articles in the English language related to FMT research were published.^[^ ^43^ ^]^ FMT has also been identified as a potential treatment for several diseases that occur beyond the gut. The concept of specific gut‐organ axes has been proposed to reflect the potential correlations in mechanisms of disease development between these organs and the gut. Well‐known examples include the gut‐brain axis (e.g. autism and depression),^[^ ^44^, ^45^ ^]^ gut‐cardiac axis (e.g. myocarditis and hypertension),^[^ ^46^, ^47^ ^]^ gut‐pulmonary axis (e.g. pneumonia),^[^ ^48^ ^]^ gut‐liver axis (e.g. non‐alcoholic steatohepatitis),^[^ ^49^, ^50^ ^]^ gut‐immune axis (e.g. systemic lupus erythematosus and graft‐versus‐host disease, GVHD),^[^ ^51^, ^52^ ^]^ and gut‐skin axis (e.g. atopic dermatitis).^[^ ^53^ ^]^ Of note, the scope of gut‐organ axes is continuously expanding, as researchers are discovering new connections between the gut microbiome and various organs in the body.

Humans must not like feces from other people and have been trying to escape this reality, but have never succeeded. The colonic transendoscopic enteral tubing (TET) was reported as a new delivery of microbiota suspension.^[^ ^54^ ^]^ It has been used to deliver microbiota for patients with severe intestinal diseases, such as CDI^[^ ^55^ ^]^ and IBD.^[^ ^24^, ^56^ ^]^ Therefore, colonic TET represents a milestone in the field of microbiota or drug delivery,^[^ ^4^ ^]^ offering a promising novel route for repeated administration throughout the colon. This innovative approach not only enhances the safety of treatments but also aligns seamlessly with physiological principles. The widespread adoption of colonic TET in China highlights its clinical versatility and the significant benefits it offers in addressing various challenging scenarios, including in situ sampling in the cecum.^[^ ^54^ ^]^ A recent animal study unveiled variations in the virome along the gastrointestinal tract, with luminal samples from the large intestine exhibiting the highest loads and diversity of bacteriophages.^[^ ^57^ ^]^ Similarly, in humans, samples obtained directly from the intestines demonstrated a higher prevalence of prophage induction compared to stool samples.^[^ ^58^ ^]^ Based on the above, colonic TET can emerge as a promising method for future investigations into the intricate interplay between the virome and hosts.

Another milestone is the drug approvals for FMT. The prepared fecal microbiota has been approved as one of the live biotherapeutic products by Australia and the United States in 2022 and 2023, respectively.^[^ ^59^ ^]^ The spores collected from donated feces also received final approval from the United States Food and Drug Administration (FDA) in 2023.^[^ ^21^ ^]^ These approved drugs play a crucial role in advancing the field of microbiota‐based therapies by facilitating their utilization in a broader population.

### 2.2 The Safety and Regulation Concerns

A systematic review^[^ ^60^ ^]^ has summarized the reported FMT‐related AEs in 5688 cases from 2000 to 2020. This review revealed that FMT‐related AEs were observed in 19% of FMT procedures, and the most frequently reported FMT‐related AEs were diarrhea (10%) and abdominal discomfort/pain/cramping (7%). Significantly, FMT‐related serious AEs have been reported in 1.4% of patients. These serious AEs include five FMT‐related deaths, attributed to fatal aspiration pneumonia related to the upper gastrointestinal delivery route (two cases had a specific causal relation, and one probably had),^[^ ^61^, ^62^, ^63^ ^]^ as well as aspiration during sedation for FMT administered via colonoscopy,^[^ ^64^ ^]^ and transmission of pathogenic bacterial species.^[^ ^65^ ^]^ A case of transmission of pathogenic bacterial species was reported in 2019,^[^ ^65^ ^]^ in which a patient developed extended‐spectrum beta‐lactamase‐producing *E. coli* bacteremia after undergoing FMT. Genomic sequencing confirmed that the causality was linked to the donor. While this was not the first case of FMT‐related death, it prompted changes in the management requirements for FMT implementation in the United States and other countries.^[^ ^66^, ^67^, ^68^ ^]^ Despite the above FMT‐related AEs having been reported, the overall safety profile of FMT is favorable.^[^ ^69^ ^]^ Although the data on the long‐term safety of FMT is insufficient, the immunogenicity of bacteria is stable in human transmission and over time.^[^ ^70^ ^]^

To further ensure the safety of FMT, it is important to focus on the methodology. Most of the published guidelines or consensus on FMT used manual methods,^[^ ^26^ ^]^ where the methodology of FMT described was still crude.^[^ ^66^, ^67^, ^68^, ^71^, ^72^, ^73^ ^]^ In a real‐world setting by Chen's group in the United States, patients who underwent FMT at academic centers differed significantly in clinical characteristics from those treated at a private practice.^[^ ^74^ ^]^ Importantly, the efficacy of FMT can be influenced by various parameters such as the manufacturing process of fecal preparation, doses, and delivery routes.^[^ ^14^, ^15^ ^]^ The heterogeneity of FMT can also lead to poor reproducibility of the research. By November 2020, a systematic review involving 30 randomized controlled trials (RCT) of FMT suggested that uniform and standardized methods were needed for research.^[^ ^75^ ^]^ Despite different microbial taxa of donors of responders and non‐responders having been observed, the inconsistent results were due to the different investigating criteria. Such inconsistencies might increase the burden of regulating FMT, leading to varied regulations and ethical considerations among countries.

By putting appropriate and effective regulations in place to safeguard patients and donors, the safety and efficacy of FMT can be improved.^[^ ^76^, ^77^ ^]^ Since 2013, the FDA has permitted the use of FMT under “enforcement discretion” for treating CDI that does not respond to standard therapy.^[^ ^78^ ^]^ In November 2022, the Australian Therapeutic Goods Administration approved the registration of BIOMICTRA, a donor‐derived microbiome‐based therapy product, for the treatment of recurrent CDI, and it has been added to the Australian Register of Therapeutic Goods. Beforehand, FMT was regarded as an investigational medicinal product in Australia.^[^ ^79^ ^]^ FMT was not approved as a drug until 2023 when the FDA granted approval to Rebyota (RBX2660).^[^ ^59^ ^]^ This microbiota‐based live biotherapeutic, prepared from human stool and consisting of a broad consortium of microbes, is indicated for the prevention of recurrence of CDI in individuals 18 years of age and older who have received antibiotic treatment for recurrent CDI. In China, FMT is a permitted medical therapy for CDI and many other diseases under the supervision of the National Health Commission.^[^ ^18^ ^]^ Significantly, in 2020, the FDA issued a warning regarding the potential risk of transferring SARS‐CoV‐2 through FMT to patients. As a precautionary measure, they recommended additional safety protocols, including excluding the donor from further donations and refraining from clinical use of any FMT product derived from stool donated by the affected donor within 4 weeks prior to their first positive test. Even though the World Health Organization (WHO) declared the COVID‐19 pandemic officially over on May 5, 2023, transitioning into a phase of long‐term management, we must remain vigilant regarding the risk of SARS‐CoV‐2 transmission through fecal material. The establishment of stool banks plays a crucial role in standardizing the donor screening process, as well as in monitoring treatment outcomes and potential side effects.^[^ ^80^ ^]^ Over 20 stool banks have been established worldwide since 2012, such as OpenBiome in America, BiomeBank in Australia, the Netherlands Donor Feces Bank, and the China Microbiota Transplantation System (FmtBank).^[^ ^7^ ^]^ Fecal microbiota bank centers have partially addressed the accessibility issues of FMT for most hospitals.

## 3 The Development of Transplanting Fecal Bacteria

### 3.1 The Methods and Delivery Routes of WMT

Since 2014, microbiota preparations using an automatic purification system (GenFMTer, Nanjing, China) have been used in China.^[^ ^51^, ^56^, ^81^ ^]^ This is followed by three rounds involving centrifugation and suspension to automatically wash the microbiota from feces (**Table** **1**). The methods of washing the microbiota are different from the traditional crude FMT process that only involves manual suspension and filtration steps to remove larger particles, fibers, and undigested food (Figure 1).^[^ ^82^ ^]^ In 2018, Zhang et al.^[^ ^5^ ^]^ proposed that it was time to discontinue manual FMT and promote standardization of the procedure. The improved methodology of FMT based on the automatic washing process and the related delivering consideration was coined as WMT,^[^ ^26^ ^]^ and released by the FMT‐standardization Study Group in 2019.^[^ ^27^ ^]^

**Table 1:** The preparation methodology for fecal transplants.

Items	WMT^[^ ^5^, ^18^ ^]^	Bacteria spores treatment^[^ ^86^ ^]^	FVT^[^ ^19^ ^]^
Conditions	GMP	GMP	Biosafety cabinet
Fecal weight	≥50 g	≥50 g	≥50 g
Origin	Allogenic	Allogenic	Allogenic
Equipment	GenFMTer	Undefined	Blender, and custom‐built air pressure filtration system
Processes	Suspension: at the ratio of 500 mL of sterile saline per 100 g of feces. Microfiltration: multi‐level automatic filtration within a disposable sterile closed‐loop system (GenFMTer). Centrifugation and washing.	Suspension: normal saline with feces. Solvent treatment: the slurry is combined with 100% ethanol to 50% (wt/wt.). Centrifugation and suspension.	Suspension: at the ratio of 500 mL of sterile saline per 50 g of feces. Centrifugation and collection of the supernatant. Filtration with different pore sizes.

GMP, good manufacturing practice.

WMT not only offers a refined methodology compared to manual FMT but also provides a safe and convenient delivery route. TET is the delivery route to address the current clinical requirements and limitations of manual FMT.^[^ ^83^ ^]^ TET involves endoscope‐assisted implantation of the tube into the deep intestine, followed by fixation. This method has great potential to adapt to the requirements of repeated transplantations.^[^ ^84^ ^]^ TET is classified into mid‐gut TET and colonic TET, depending on the implantation location in the intestine. The colonic TET can reduce the spatial and temporal heterogeneity of different studies because the implantation site can be precisely identified and the sampling time can be standardized.^[^ ^85^ ^]^ The improved quantitative methods, automatic purification systems, and delivery approach of WMT have led to reduced research heterogeneity and improved safety.

### 3.2 The Efficacy and Safety of Washed Microbiota Transplantation

Two previous studies^[^ ^24^, ^56^ ^]^ have reported that WMT is an independent factor that reduces AEs in patients with ulcerative colitis (UC) and Crohn's disease (CD). The latest study^[^ ^18^ ^]^ updated the safety data of WMT and compared it with manual FMT. The incidence of AEs decreased from 35.5% (11/31) of manual FMT to 7.2% (65/902) of WMT in patients with UC, and from 21.7% (15/69) of FMT to 4% (35/882) of WMT in patients with CD. An animal study^[^ ^26^ ^]^ explained the improved safety of WMT could be attributed to the repeated washing process, which helps to remove a growing number of pro‐inflammatory metabolites (such as leukotriene B4, corticosterone, and prostaglandin G2), and viruses.

Intriguingly, fresh WMT achieved the same duration of remission in a patient with refractory UC with less frequent treatments compared to fresh FMT (**Table** **2**).^[^ ^23^ ^]^ In this study, the patient achieved the same duration of eight‐month remission with only two courses of WMT via colonic TET, while the same duration was achieved with 60 times of FMT via a percutaneous endoscopic cecostomy tube. In clinical practice, reducing both the frequency of treatment and the length of hospital stays can contribute to increasing patients' compliance. Although the biomarkers of responsiveness to WMT for IBD are unclear, decreasing *Candida* abundance has been positively correlated with the amelioration of disease severity.^[^ ^87^ ^]^ Patient with refractory UC suffering from recurrent fungal infection has also been successfully cured by WMT (Table 2).^[^ ^88^ ^]^ Within one week after the initial WMT, the *C. glabrata* infection in fecal fungal culture turned negative.

**Table 2:** Summary of clinical and animal studies into transplanting bacteria or virome.

Disease	Study types	Subjects	Donors	Delivery routes	Outcomes
Transplanting bacteria					
CDI	Double‐blind, randomized, placebo‐controlled study^[^ ^21^ ^]^	182 patients (SER‐109 (*N* = 89), placebo (*N* = 93))	Healthy individuals	Capsule	Recurrence rates of CDI were significantly lower in the SER‐109 group (12%) compared to the placebo group (40%). Most AEs were mild to moderate and similar in both groups.
UC	Real‐world study^[^ ^56^ ^]^	109 patients	Healthy individuals	Colonic TET; mid‐gut TET	74.3% (81/109) and 51.4% (56/109) of patients achieved clinical response at 1 month and 3 months after WMT, respectively; and 25.7% (28/109) and 20.2% (22/109) of patients achieved clinical remission at 1 month and 3 months after WMT, respectively. During the follow‐up period of one–five years, 17.4% of AEs occurred. No AE beyond one month was observed.
	Case report^[^ ^23^ ^]^	One patient with refractory UC	Healthy individuals	Colonic TET; gastroscopy	The patient underwent 60 FMTs via percutaneous endoscopic cecostomy tube in two months and achieved clinical remission for eight months. Then the patient underwent seven courses of WMT (a total 10 WMTs, 8 by colonic TET). After two courses of WMT, the patient achieved clinical remission for eight months. The total remission time induced and maintained by the last two courses of WMT added up to 45 months.
	Case report^[^ ^88^ ^]^	One patient with refractory UC complicated with recurrent fungal infection	Healthy individuals	Nasojejunal tube	The patient was diagnosed with UC (E3, severe activity, Mayo score = 11), C. glabrata infection, adrenocortical insufficiency, osteoporosis, femoral head necrosis, psoriasis, Hashimoto thyroiditis, and moderate malnutrition. After five times of WMTs and then plus cyclosporine, the stool frequency decreased from 10–15 times per day to twice per day 2 weeks later. The fecal fungus culture remained negative 3 and 6 months after the first WMT.
CD	Real‐world study^[^ ^24^ ^]^	139 patients	Healthy individuals	Mid‐gut TET	During one month after WMT, 13.6% of mild AEs occurred. No AE beyond one month was observed. The rate of clinical response and clinical remission was 45% (9/20) and 20% (4/20) in the patients with AE, and 75.6% (90/119) and 63.0% (75/119) in the group without AE.
Dyslipidemia	Real‐world study^[^ ^98^ ^]^	177 patients (40 patients with hyperlipidemia, 87 patients with normal blood lipids, and 50 patients with hypolipidemia)	Healthy individuals	Nasojejunal tube; Colonic TET	In the hyperlipidemia group, hyper‐blood lipid decreased to normal within 33–47 days (35.14%; *p* <0.001), and LDL‐C changed to normal within 34–63 days (33.33%; p = 0.013) after WMT. In the hypolipidemia group, 36.36% and 47.06% changed to normal within 33–47 days (*p* = 0.006) and 34–63 days (*p* = 0.005) after WMT. In the normal blood lipid group and the low‐risk group of atherosclerotic cardiovascular disease, the change was not statistically significant after WMT.
High blood glucose	Real‐world study^[^ ^89^ ^]^	195 patients (20 patients with high blood glucose and 175 patients with normal blood glucose)	Healthy individuals	Nasojejunal tube; Colonic TET	After WMT, the fasting blood glucose of 72.22% of patients with high blood glucose decreased to normal within ≈1 month (*p* < 0.001). Within ≈ 2 months, there was a significant hypolipidemic (*p* = 0.043) effect. Within ≈ 6 months, there was a significant blood pressure lowering (systolic blood pressure, *p* = 0.048) effect.
Diabetes	Animal study plus a randomized, double‐blind, and placebo‐controlled trial^[^ ^90^ ^]^	Animal study one: mice (M)‐normal glucose (NG) group (*n =* 13), M‐diabetes mellites (DM) group (*n =* 13), M‐distal symmetric polyneuropathy (DSPN) group (*n =* 13). Animal study two: M‐NG group (*n =* 6), and M‐DSPN group (*n =* 6) RCT: WMT group (*n =* 22), and placebo group (*n =* 10).	Animal study: healthy individuals, patients with DM but not DSPN, and patients with DSPN. RCT: healthy individuals.	Animal study: oral gavage. RCT: mid‐gut TET.	Animal study: The fecal transplantation from patients with diabetes complicating distal symmetric polyneuropathy to mice resulted in the transfer of diabetes‐related phenotypes. RCT: After 84 days, several positive outcomes were observed in the group that underwent WMT compared to the placebo group. These included improvements in neuropathic symptoms, neuropathic pain, anxiety levels, sleep quality, and electrophysiological functions of peripheral nerves. Notably, the relief of moderate to severe neuropathic pain showed a significant difference between the two groups, with 53.3% experiencing relief in the WMT group compared to only 14.29% in the placebo group. Additionally, sensory nerve conduction velocities were significantly increased in the WMT group.
Hypertension	Real‐world study^[^ ^96^ ^]^	260 patients (73 hypertensive patients and 187 normotensive patients)	Healthy individuals	Nasojejunal tube; Colonic TET	After WMT, the blood pressure at hospital discharge was significantly lower than that at hospital admission (change in systolic blood pressure: −5.09 ± 15.51, *p =* 0.009; change in diastolic blood pressure: −7.74 ± 10.42, *p* < 0.001). Hypertensive patients not taking antihypertensive drugs also had a greater decrease in systolic (β = −8.969, standard error = 4.256, *p =* 0.040) and diastolic blood pressure (β = −8.637, standard error = 2.861, *p =* 0.004).
ALS	Case report^[^ ^99^ ^]^	One patient	Healthy individuals	Colonic TET; mid‐gut TET	Constipation and ALS symptoms such as impaired balance and gait were well controlled since the first course of WMT. Although the patient was administered antibiotics after several months because of an accidental fall and then her muscle tone returned to her worst status, the rescue WMT successfully stopped the progression of the disease again with quick improvement. Microbial analysis indicated that the diversity and composition of the gut microbiota from the patient after WMT treatment were closer to healthy donors.
ASD	Real‐world study^[^ ^101^ ^]^	42 child patients	Healthy individuals	Colonic TET	Aberrant Behavior Checklist, Childhood Autism Rating Scale, and Sleep Disturbance Scale for Children scores, the proportion of children with constipation and abnormal fecal forms, and WBC and globulin levels were all significantly lower in ASD children after WMT.
Antibiotic‐associated dysbiosis	Real‐world study^[^ ^104^ ^]^	18 critically ill patients	Healthy individuals	Nasojejunal tube; gastroscopy; enema	One hundred percent (2/2) of abdominal pain, 86.7% (13/15) of diarrhea, 69.2% (9/13) of abdominal distention, and 50% (1/2) of hematochezia were improved after WMT. 44.4% (8/18) of patients recovered from abdominal symptoms without recurrence and survived for a minimum of 12 weeks after being discharged from the ICU. 38.9% (7/18) of patients had WMT‐related AEs during follow‐up, including increased diarrhea frequency, abdominal pain, increased serum amylase, and fever.
Radiation enteritis	Real‐world study^[^ ^105^ ^]^	5 patients	Healthy individuals	Mid‐gut TET; gastroscopy	3/5 patients responded to WMT which was defined as a 1‐grade reduction in Radiation Therapy Oncology Group (RTOG/EORTC) late toxicity grade from baseline by 8 weeks after WMT. No WMT‐induced death and infectious complications were observed. One mild AE occurred in case 4, who experienced transient nausea immediately after WMT and self‐resolved within 24 hours.
GVHD	Real‐world study^[^ ^51^ ^]^	8 patients	Healthy individuals	Nasoduodenal tube	All the patients achieved clinical symptomatic remission after the first WMT. Among patients with diarrhea, their stool volumes and frequencies were reduced to 3–4 times/day after WMT. In the 2‐week follow‐up after the first WMT, two patients' diarrhea and all the other five patients' abdominal pain disappeared (three patients died within this period unrelated to WMT). Compared to those who did not receive WMT, these eight patients achieved a higher progression‐free survival.
Transplanting virome					
CDI	Real‐world study^[^ ^19^ ^]^	5 patients	Healthy individuals	nasojejunal tube	In all 5 patients, FVT restored normal stool habits and eliminated symptoms of CDI for a minimum period of 6 months. Bacterial phylogeny and virome profile analyses of fecal samples from recipients indicated longitudinal changes in microbial and viral community structures after FVT.
T2D and obesity	Animal study^[^ ^20^ ^]^	40 mice were divided into 5 groups including a low‐fat diet (as lean control), high‐fat (HF) diet, HF+ampicillin, HF+ ampicillin +FVT, and HF+FVT	C57BL/6N mice fed LF diet for 14 weeks	oral gavage	Six weeks after the first FVT from lean donors, A decreased weight gain and a normalized blood glucose tolerance in a diet‐induced obesity mouse model were observed. FVT significantly changed the bacterial and viral gut microbiota component, as well as the plasma metabolome and the expression profiles of obesity and T2D‐associated genes.
SIBO	Animal study^[^ ^106^ ^]^	36 mice divided into two dietary groups (*n =* 18 each): standard diet (SD) or high‐fat diet (HFD)	18 mice fed HFD for 30 days	oral gavage	In both recipient groups, FVT produced similar results to the whole FMT. FVT did not significantly alter the population density of resident small intestinal bacterial community composition in SD recipients. However, it did induce a transition in the small intestinal bacterial composition, making it resemble that of an HFD microbiome. Following FVT, the bacterial density in the ileum of recipients on an HFD was reduced.
Antibiotic‐associated dysbiosis	Animal study^[^ ^107^ ^]^	16 post‐antibiotic treated mice were divided into two groups: FVT (*n =* 8) or heat‐ and nuclease‐treated FVT as a control (*n =* 8).	16 mice before antibiotic treatment	oral gavage	An autochthonous virome transfer reshaped the bacterial communities of mice following antibiotic treatment, resulting in a microbiota profile that closely resembled the pre‐antibiotic state. This effect was not observed in mice that received non‐viable phages. Metagenomic sequencing of the virome showed that the abundance and diversity of fecal bacteriophages differed over time between the FVT group and the control group. Notably, the phages introduced through FVT persisted in the mice that received them.
NEC	Animal study^[^ ^108^ ^]^	59 preterm, cesarean‐delivered piglets divided into four groups: rectal FMT administration (*n =* 16), rectal FVT (*n =* 14), oro‐gastric administration (*n =* 13), and saline (*n =* 16)	healthy suckling piglets	Enema; oral gavage	Oro‐gastric FVT completely prevented NEC, which was confirmed by microscopy, whereas FMT did not perform better than the control. Oro‐gastric FVT increased viral diversity and reduced Proteobacteria relative abundance in the ileal mucosa relative to control. Induction of mucosal immunity was observed in response to FMT but not FVT. Oro‐gastric FVT also performed better than FMT in a series of safety parameters, including body growth rate, the relative weight of the small intestine, small intestinal permeability, and mucosal integrity.

CDI, *Clostridiodes difficile* infection. UC, ulcerative colitis. CD, Crohn's disease. ALS, amyotrophic lateral sclerosis. ASD, autism spectrum disorder. GVHD, graft‐versus‐host disease. SIBO, small intestinal bacterial overgrowth. NEC, necrotizing enterocolitis.

In metabolic diseases, the latest studies have demonstrated the efficacy and safety of WMT in improving diabetes.^[^ ^89^, ^90^ ^]^ The animal study has observed that fecal transplantation from patients with diabetes complicating distal symmetric polyneuropathy to mice could lead to the transfer of diabetes phenotypes. This transfer may be attributed to several potential mechanisms, including a compromised gut barrier, elevated antigen load, and systemic inflammation. Specifically, lipopolysaccharide (LPS) was able to breach the compromised gut barrier and enter the bloodstream. Subsequently, LPS induced the production of pro‐inflammatory cytokines such as IL‐1β, IL‐6, IL‐8, and TNF‐α in a Toll‐like receptor 4‐dependent manner, thereby promoting inflammation and exacerbating damage to islet β cells, consequently accelerating the progression of diabetes.^[^ ^91^ ^]^ In the RCT segment of the same study, it was demonstrated that frozen WMT could improve neuropathic pain in patients. The WMT group showed a significant improvement in neuropathic pain compared to the placebo group (WMT group: 53.3% vs. placebo group: 14.29%).^[^ ^90^ ^]^ This improvement following WMT could potentially be attributed to an increase in the gut microbiota's butyric acid production capacity and a decrease in LPS production. Another study reported that 72.22% of patients with high blood glucose restored their fasting blood glucose to normal within 1 month after fresh WMT.^[^ ^89^ ^]^ In addition, long‐term (≈6 months) effects on lowering blood pressure after WMT have also been shown in patients with high blood glucose (Table 2).^[^ ^89^ ^]^ Previous studies in animal models have suggested that gut microbiota play a role in regulating blood pressure.^[^ ^92^, ^93^ ^]^ For example, hypertensive models such as SHRs and Ang II mice have fewer SCFA‐producing bacteria than normotensive mice.^[^ ^94^ ^]^ Decreased SCFA could cause neuroinflammation, further affecting blood pressure.^[^ ^95^ ^]^ In clinical practice, a recent study indicated that fresh WMT had a short‐term antihypertensive effect on patients with hypertension (Table 2).^[^ ^96^ ^]^ Compared with baseline, patients with hypertension after WMT had an increased abundance of *Senegalimassilia* species and a decreased abundance of *Parasutterella* and *Solobacterium* species. Among these species, *Solobacterium* species has been proven to have an association with atherosclerotic cardiovascular disease.^[^ ^97^ ^]^ Another study involving patients with dyslipidemia showed that 35.14% of patients with hyperlipidemia returned to normal blood lipid levels one month after fresh WMT.^[^ ^98^ ^]^ In the hypolipidemia group,^[^ ^98^ ^]^ 36.36% and 47.06% became normal in one and two months after WMT, respectively (Table 2).

A recent case report presented clinical evidence of the effectiveness of WMT in halting the progression of amyotrophic lateral sclerosis (ALS), and trends in the microbiome and metabolome were consistent with improvements in disease status (Table 2).^[^ ^99^ ^]^ Similarly, in individuals with ASD, alterations in the composition of the microbiota have been recognized by researchers as a key feature of this pervasive developmental disorder.^[^ ^100^ ^]^ A retrospective study of children with ASD has demonstrated that symptoms of ASD, gastrointestinal issues, sleep disorders, and systemic inflammation can be improved with fresh WMT (Table 2).^[^ ^101^ ^]^ The potential mechanism might be attributed to that WMT could mitigate the production of p‐cresol sulfate and 5‐hydroxytryptamine, which are associated with microbiota dysbiosis.^[^ ^102^, ^103^ ^]^

**Figure 2:**
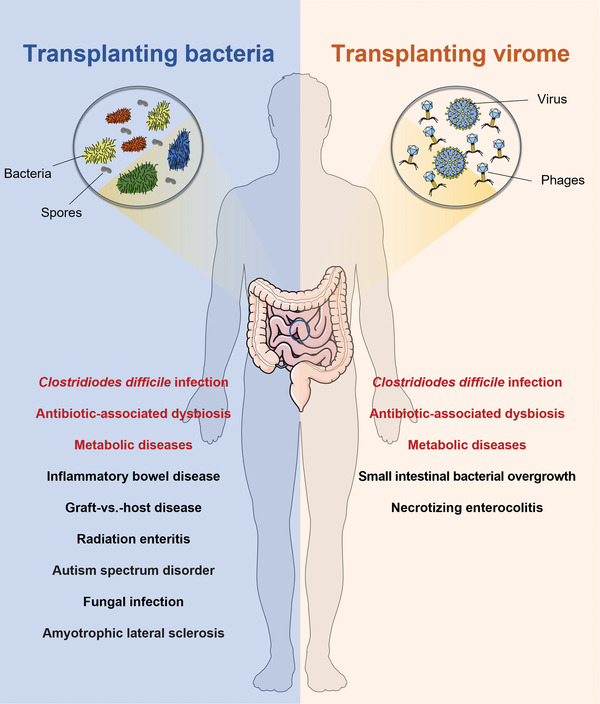
The applications of transplanting bacteria or virome. Applications that have clinical or preclinical evidence of transplanting bacteria or virome are listed on the corresponding side. If an application has evidence on both sides, it is highlighted in red.

The methodology of WMT has been widely used in China since 2014, as described in the studies on the treatment of antibiotic‐associated diarrhea,^[^ ^104^ ^]^ radiation enteritis,^[^ ^105^ ^]^ and GVHD (Table 2).^[^ ^51^ ^]^ As mentioned above, WMT has shown its efficacy and safety in treating numerous diseases (**Figure** **2**), providing researchers with confidence in its potential.

### 3.3 The Methodology and Delivery Routes of Bacteria Spores Treatment

Another method to enrich bacteria for transplanting is by purifying bacteria spores from fecal matter.^[^ ^25^ ^]^ Bacteria spores are a means of survival in hostile environments, such as those created by antibiotic treatments. This presents a significant challenge for clinicians, as antibiotics cannot kill spores.^[^ ^109^ ^]^ However, it has been observed that utilizing nontoxigenic bacteria spores that compete with toxigenic spore‐forming bacteria has the potential to solve this challenge.^[^ ^21^, ^110^ ^]^ The methods of purifying nontoxigenic bacteria spores (Figure 2) involve solvent treatments (a combination of freeze‐thaw in 50% ethanol followed by 2 h in 70% ethanol at room temperature) and purification steps (pelleting by centrifugation, washing with saline to remove ethanol, resuspending with sterile glycerol) to clear vegetative bacteria, fungi, parasites, and viruses (Table 1).^[^ ^25^, ^86^ ^]^ Following these processes, patients are given capsules containing purified bacteria spores through oral administration.^[^ ^25^, ^86^ ^]^

### 3.4 The Efficacy and Safety of Bacteria Spores Treatment

Purifying bacteria spores has been primarily used in the treatment and prevention of recurrent CDI (Figure 2).^[^ ^21^, ^110^ ^]^ *C. difficile* is a Gram‐positive, spore‐forming, toxin‐producing, obligate anaerobic bacterium. The spore coat is a physical barrier that protects *C. difficile* against chemical insults from hosts and natural environments. *C. difficile* spores are resistant to antibiotics and can rapidly germinate into vegetative bacteria after antibiotic treatment is stopped.^[^ ^109^ ^]^ In 2015, Gerding et al.^[^ ^110^ ^]^ reported that administration of spores from nontoxigenic *C. difficile* strain M3 could prevent recurrent CDI. They hypothesized that nontoxigenic *C. difficile* strain M3 could compete for the same metabolic or adherence niche in the gastrointestinal tract with toxigenic *C. difficile*. Recently, an investigational oral microbiome therapeutic called SER‐109,^[^ ^21^, ^86^ ^]^ composed of donor's live purified Firmicutes bacterial spores from ≈ 34 different genera, has been developed to compete metabolically with *C. difficile* for essential nutrients and modulate bile‐acid profiles to reestablish resistance to colonization by *C. difficile*. Purified Firmicutes bacterial spores have shown high efficacy and safety in treating CDI (Table 2).^[^ ^111^ ^]^ In April 2023, SER‐109 (tradename: Vowst) received FDA approval as an oral microbiota drug, with the indication for the prevention of recurrent CDI. Because SER‐109 is considered to restore the balance of gut microbiota rather than killing *C. difficile*. Although the compositions of practical spores may vary among donors, the improved methodology involving solvent treatments and purification steps can increase safety without relying solely on donor screening.^[^ ^21^ ^]^ Besides, oral administration and capsule preparation facilitate quantification of the therapeutic dose,^[^ ^25^ ^]^ which can help reduce research heterogeneity.

### 3.5 The Future of Transplanting Fecal Bacteria

Although studies have shown that enriching the bacteria from feces did not reduce the efficacy of WMT,^[^ ^24^, ^56^ ^]^ it is worth noting that some substances, such as viruses, microbial secretions, and metabolites, have been mostly washed out. Exploring more precise bacteria for transplanting is an emerging trend in the development of microbiota‐based therapies. Strain level diversity and complementarity are considered to be the strongest determinants of FMT results,^[^ ^16^ ^]^ and more accurate sequencing strategies would contribute to evaluating the efficacy or selecting the most suitable enriched bacteria for patients.^[^ ^112^, ^113^ ^]^ Live biotherapeutic products consisting of defined bacterial consortia, such as VE303, have also demonstrated stable and effective results.^[^ ^36^, ^114^ ^]^ However, it may eliminate certain fecal components whose functions are not yet fully understood but are believed to play crucial roles. Although existing sequencing techniques are adequate for identifying the organisms present in the gut microbiota and inferring their functional potential, they fall short in pinpointing which species are more active and their specific functional contributions within the human body. To address this limitation, mature multiomics sequencing strategies, such as the introduction of metatranscriptome analysis, can be employed.^[^ ^115^ ^]^ Besides, the human gut microbiome is a highly complex and heterogeneous ecosystem, with significant variations even within the same individual's microbiota.^[^ ^116^ ^]^ Careful attention to experimental design, including refined sample collection processes with reduced variation, and enhanced data analysis techniques such as absolute microbiome measurements, can also contribute to overcoming the challenges associated with certifying the efficacy of defined bacterial consortia across a large and diverse population.^[^ ^117^ ^]^

## 4 The Development of Transplanting Fecal Virome

### 4.1 The Methodology and Delivery Routes of FVT

Viral therapy can date back to at least the 20th century, as bacteriophages were discovered in 1915^[^ ^118^ ^]^ and their use in treating dysentery was reported shortly after.^[^ ^119^ ^]^ Among human gut bacteriophages, the tailed double‐stranded DNA Caudovirales order and the single‐stranded DNA *Microviridae* family are the most common. As the main vector of horizontal gene transfer, bacteriophages can strongly influence bacterial evolution, diversity, and metabolism.^[^ ^120^ ^]^ Therefore, bacteriophages play a key role in shaping the community structure and stability of human gut microbiota.^[^ ^35^ ^]^ In recent years, researchers have found that the gut virome is associated with several diseases, including recurrent CDI,^[^ ^121^ ^]^ IBD,^[^ ^122^, ^123^ ^]^ metabolic syndrome,^[^ ^124^ ^]^ GVHD,^[^ ^125^ ^]^ and even cancer.^[^ ^126^ ^]^ One angle of research supporting the role of the virome is the association between efficacy and overlapped viral communities. Studies^[^ ^17^, ^121^, ^127^ ^]^ of recurrent CDI indicated that recipients who benefited from FMT had an increased relative abundance of *Microviridae* and a decreased abundance of Caudovirales. These results suggested that the *Microviridae* family and Caudovirales order potentially played a pivotal role in the efficacy of FMT. Moreover, Broecker et al.^[^ ^128^ ^]^ observed that stable bacteriophages remained present for at least 4.5 years following FMT and were better correlated with successful FMT than bacterial communities. The negative correlation between recovery of the virome and CDI recurrence can further certify the vital role of the virome in FMT. For example, one study^[^ ^121^ ^]^ reported that CDI patients who only had restored bacterial communities suffered from disease recurrence. In subjects with metabolic syndrome who did not respond to FMT, the differences between the viral communities shared by the recipient and the respective donor were greater.^[^ ^124^ ^]^

Another angle that demonstrates the potential of the virome is the difference in virome characteristics between disease and health. Several studies have shown an increasing richness of Caudovirales in patients with IBD compared to healthy participants.^[^ ^123^, ^129^, ^130^ ^]^ Furthermore, a recent study^[^ ^131^ ^]^ indicated that transplanting the virome of UC patients into mice could exacerbate the severity of DSS colitis, leading to shortening colon length and increasing pro‐inflammatory cytokine production such as TNF‐α and IL‐1β. This study excluded the interference of endotoxin in the virome preparations, such as lipopolysaccharide, because mice given heat‐killed virome displayed reduced colitis severity compared to those given intact virome of UC patients.^[^ ^131^ ^]^ Gut virome also relates to environmental factors, such as diet^[^ ^132^ ^]^ and antibiotic exposure,^[^ ^133^ ^]^ which are known as risk factors for IBD. While the previous studies have primarily focused on the role of the bacterial microbiome,^[^ ^134^, ^135^ ^]^ recent research has identified associations between enteric GVHD and viruses from the families *Herpesviridae* and *Adenoviridae*.^[^ ^136^, ^137^, ^138^ ^]^ GVHD is a serious complication that is one of the most common causes of death arising from allogeneic hematopoietic stem cell transplant.^[^ ^139^ ^]^ Immunosuppressants are typically used to treat GVHD, and a study found a higher presence of eukaryotic viruses relative to bacteriophages in the GVHD patient before FMT,^[^ ^125^ ^]^ which may be connected to immunosuppressant use.^[^ ^140^ ^]^ Compared to individuals without GVHD after allogeneic hematopoietic stem cell transplantation, enteric GVHD patients have exhibited a decrease in phage richness and a continuous increase in the number and detection rate of DNA viruses (*Anelloviridae*, *Herpesviridae*, *Papillomaviridae*, and *Polyomaviridae*).^[^ ^141^ ^]^ In the latest meta‐analysis, viruses mainly associated with butyrate‐producing bacteria were found to be reduced in patients with colorectal cancer.^[^ ^126^ ^]^ Butyrate‐producing bacteria, such as *F. prausnitzii*, have anti‐tumorigenic properties and might contribute to preventing colorectal cancer development.^[^ ^142^ ^]^ Hence, differences in the virome may serve as cancer‐associated viral markers to guide future practice.

FVT has emerged as a potential alternative to FMT. Unlike transplanting the entire fecal microbiome, FVT focuses on transplanting the virome in the feces of donors. To prepare the fecal virome, bacteria are removed from the donor feces through a sterile preparation process that employs filters with pore sizes smaller than bacteria (Table 1).^[^ ^19,35^ ^]^ This allows for the retention of the virome while removing potentially harmful bacteria. Accordingly, FVT has the potential to decrease the risk of invasive bacterial infections.^[^ ^17^, ^143^ ^]^ Of note, the delivery route of FVT varied in different studies, such as nasojejunal tube,^[^ ^19^ ^]^ orogastric,^[^ ^131^ ^]^ and enema (Table 2).^[^ ^144^ ^]^ This variability could be attributed to the fact that FVT is still in the exploratory stage and has yet to be widely adopted in clinical practice.

### 4.2 The Efficacy and Safety of FVT

In 2017, a preliminary investigation mentioned that the fresh sterile fecal filtrates (with fecal virome) could alleviate symptoms of patients with CDI (Table 2).^[^ ^19^ ^]^ After FVT, they restored normal stool habits and experienced the elimination of CDI symptoms for at least 6 months. Although this study had a small sample size and was non‐randomized, the significant benefits to patients gave researchers confidence to study FVT further.

In metabolic diseases, a mice study^[^ ^20^ ^]^ demonstrated that FVT induced the reduction of symptoms of type 2 diabetes (T2D) and obesity. They observed that the reduced Shannon Diversity Index of the bacterial community in mice that were fed an HFD could be restored to normal levels after receiving FVT from lean donors. Besides, they found increasing gene expressions such as Lepr, Klb, Ppargc1a, and Igfbp2 in ileum tissue (Table 2). Importantly, previous studies^[^ ^145^, ^146^, ^147^, ^148^ ^]^ have proved a positive correlation between all of the above genes with protection against T2D or lower body weight. A recent study yielded similar results, showing that virome‐induced perturbations could modify the fecal microbiome in vivo, and this alone was adequate to induce both lean and obese phenotypes in mice.^[^ ^149^ ^]^ Moreover, a high‐fat diet not only increases the risk of obesity and T2D but also disrupts the composition and function of the gut microbiota, leading to a range of gastrointestinal disorders including small intestinal bacterial overgrowth (SIBO). In a study conducted on mice,^[^ ^150^ ^]^ it was observed that after being on a high‐fat diet for 30 days, the intervillous zone of the ileum was occupied by a dense microbiota, which is a characteristic of SIBO.^[^ ^151^ ^]^ Another study^[^ ^106^ ^]^ created a mice model of SIBO by feeding them a high‐fat diet. These mice became donors and provided fecal transplants for mice on a standard diet or high‐fat diet. The researchers found that both FMT and FVT could convert the gut microbiota of healthy mice into high‐fat diet‐related components. Intriguingly, although FMT and FVT had similar effects on the recipients, mice fed with different diets had different results. The bacterial density of recipients on a high‐fat diet reduced, while those on a standard diet did not (Table 2). This result suggests that both FMT and FVT could have similar effects on normalizing the mice with SIBO. SIBO has been identified in up to 78% of IBS patients.^[^ ^152^, ^153^ ^]^ Antibiotics are typically used as the first‐line treatment for SIBO. However, a study showed that 43.7% (35/80) of SIBO patients experienced a relapse 9 months after completing antibiotic treatment.^[^ ^154^ ^]^ Therefore, there is a need for alternative therapies, and FVT may have the potential to be one such therapy. Although other studies did not directly involve the treatment of certain diseases, they demonstrated the potential of FVT in replicating the effects of certain diets. One such study explored the effects of FMT or FVT, prepared from mice fed with green tea polyphenol, on mice with DSS‐induced colitis.^[^ ^155^ ^]^ The study revealed that both FMT and FVT could ameliorate DSS‐induced colitis, as indicated by decreased inflammation factors in the plasma and improved symptoms. However, the degree of improvement observed after FMT was better than that of FVT. In another study, it was found that weight gain in mice, induced by feeding risperidone, could be achieved by FVT prepared from mice fed with risperidone.^[^ ^156^ ^]^ Furthermore, a recent study demonstrated that FVT derived from mice with a high relative abundance of *A. muciniphila* could enhance the proliferation of commensal gut *A. muciniphila* in the recipients. Surprisingly, this also resulted in an improvement in fertility rates.^[^ ^157^ ^]^ However, the underlying mechanism behind this effect warrants further investigation.

Strategies are also needed to address gut microbiota dysbiosis caused by broad‐spectrum antibiotics.^[^ ^158^ ^]^ An animal study^[^ ^107^ ^]^ showed that autochthonous FVT (collected and frozen before antibiotic treatment) can reshape the gut microbiota after antibiotic treatment (Table 2). After 15 days of initial gavage, the mice that received FVT showed less difference in abundant taxa (14% of fecal OTUs and 36% of cecally derived) when compared to the initial microbiota, whereas the difference was 87% and 50% in mice treated with heat‐ or nuclease‐treated FVT, respectively. This study suggested the potential of FVT in preventing gut microbiota dysbiosis following the use of antibiotics.

FVT might be effective for preventing necrotizing enterocolitis (NEC) in preterm infants.^[^ ^108^ ^]^ NEC is a tough challenge to clinicians because of its high frequency, lethality, and unpreventable characteristics in preterm infants.^[^ ^159^ ^]^ A study conducted on preterm piglets showed that collecting fecal materials from healthy suckling piglets and using FVT with frozen virome to prevent NEC in preterm piglets was effective.^[^ ^108^ ^]^ After stomatogastric FVT, the macroscopic and microscopic incidence of NEC reduced to 0% and lower than 20%, respectively. However, rectally administered FMT failed to reduce the severity and incidence of NEC compared to control groups (Table 2). The recognized microbiota feature of NEC in neonatal units is increased Proteobacteria and reduced Bacteroidetes relative abundances.^[^ ^144^ ^]^ Interestingly, stomatogastric FVT increased viral diversity and decreased Proteobacteria relative abundance in the ileal mucosa, highlighting the potential of FVT as a promising intervention for NEC prevention in preterm infants.^[^ ^108^ ^]^ Importantly, in this study, there was no significant alteration observed in gene expressions, suggesting that the virome did not have a substantial impact on host immune cells to induce mucosal immunity. This finding contrasts with the previously discussed study that FVT could improve T2D by altering tissue gene expression.^[^ ^20^ ^]^

### 4.3 The Future of Transplanting Fecal Virome

Although transplanting the fecal virome has shown significant potential in treating some diseases (Figure 2), the concept is still in its early stages. Bacteriophages, which are predominant in gut virome, may be sufficient for contributing to the efficacy of transplanting virome,^[^ ^160^, ^161^ ^]^ but the contribution of metabolites, proteins, and bacterial fragments in the sterile filtration cannot be excluded.^[^ ^162^, ^163^ ^]^ There are two possible explanations for the effectiveness. One is that bacterial cell wall components or DNA fragments make efforts by stimulating host response.^[^ ^162^, ^163^ ^]^ Another is the interaction of bacteriophages and gut microbiota, resulting in the resolution of initial gut dysbiosis.^[^ ^160^, ^161^ ^]^ Of note, bacteriophages rely on bacteria as hosts to function.^[^ ^164^, ^165^ ^]^ They can either destroy the bacteria (virulent or lysogenic phages) or modify their behavior (lysogenic phages), including drug resistance and virulence. These phages show promise in combating pathogen‐led diseases like CDI and antibiotic‐resistant bacterial infections.^[^ ^166^, ^167^ ^]^ They can also help regulate the intestinal environment without the need for excessive microbiota supplementation.^[^ ^168^ ^]^ However, their effectiveness may be limited in diseases caused by microbiota dysbiosis. Moreover, there is a risk of exacerbating gut microbiota disorders by introducing inappropriate viral groups. Many pathogenic eukaryotic viruses exist in the human gut, including papillomaviruses, herpes viruses, hepatitis viruses, bocaviruses, enteroviruses, rotaviruses, and sapoviruses.^[^ ^169^ ^]^ Norovirus transmission to patients with CDI via FMT has been reported because these patients developed post‐FMT norovirus gastroenteritis.^[^ ^170^ ^]^ Besides, some unwanted phage‐encoded genes may be transferred by bacteriophages,^[^ ^128^ ^]^ which can induce antibiotic resistance or bacterial virulence. For example, Bacteroidetes, Firmicutes, Verrucomicrobia, and Fusobacteria gained antimicrobial peptide resistance amiABC after FMT by inserting these lysogenic phage genomes into their genome.^[^ ^17^ ^]^

The characteristics of phages suggest that the development of the transplanted virome is closely intertwined with transplanted bacteria. This implies that the ongoing research directions focused on transplanted bacteria have the potential to shed light on the role of the virome as well. However, there is still a need for significant advancements in terms of efficacy, mechanisms, sequencing identification, and other technical aspects related to virome. Microbiota medicine, as an emerging clinical medicine discipline,^[^ ^39^ ^]^ can integrate various microbiome‐related technologies with FMT as the core, and employ education as the core force to mitigate the challenges faced by FVT.

## 5 The Sequencing and Big‐Data Analysis Technology for Microbiome

Microbiome research has rapidly advanced due to the widespread application of next‐generation sequencing (NGS) technology, which enables the discovery and characterization of microbes at a large scale. 16S rRNA sequencing,^[^ ^171^ ^]^ shotgun metagenomic sequencing (for all the DNA),^[^ ^172^ ^]^ and RNA sequencing^[^ ^173^ ^]^ can be used to study the mechanism of transplanting bacteria. Importantly, 16S rRNA sequencing cannot detect strain‐level changes while shotgun metagenomic sequencing and RNA sequencing can.^[^ ^174^ ^]^ Based on strain‐level microbiota profiling, big‐data analysis can identify core gut microbiota with the metagenomics quality control measure.^[^ ^175^, ^176^, ^177^ ^]^ Compared with bacteriome, virome requires a higher standard of sequencing technologies because of the amplified background noise interference and lower annotated rate of virome.^[^ ^178^ ^]^ Shotgun metagenomic sequencing and RNA sequencing can study the mechanism of transplanting the virome (DNA and RNA virus, respectively).^[^ ^172^, ^173^ ^]^ However, DNA or RNA virus alone cannot represent the whole virome. Besides, the characteristics of NGS, such as short read length and long sequencing time,^[^ ^179^ ^]^ would provide both biased and fragmentary knowledge of virome. Oxford Nanopore Technology (ONT) is one of the single‐molecule real‐time (SMRT) sequencing technologies.^[^ ^180^ ^]^ The improved sequencing can provide genome‐length reads that cover the entire mutation within a single virus particle,^[^ ^181^ ^]^ and information on epigenetic modifications.^[^ ^182^ ^]^ A complete working protocol has been established to profile gut virome using physical enrichment, reverse transcription, random amplification, and eventually the SMRT platform of ONT.^[^ ^181^ ^]^ Besides, hybrid and ultra‐deep metagenomic sequencing represent crucial sequencing strategies for providing a comprehensive understanding of the genomic and functional aspects of the virome.^[^ ^183^, ^184^ ^]^ As sequencing technology continues to advance, it will lead to an exponential increase in data, commonly referred to as big data, making it imperative to develop robust methods for processing and analyzing this wealth of information. These analytical strategies for handling big data have the potential to greatly enhance our exploration of the intricate interactions between the gut microbiome and the human body.^[^ ^185^ ^]^ Moreover, they hold the promise of enabling precision medicine through the microbiome, encompassing precise drug selection,^[^ ^186^ ^]^ and tailored approaches to cancer treatment,^[^ ^187^ ^]^ among other medical applications.

## 6 Allogenic or Autologous Transplantation

Almost all bacteria and viromes used for transplantation are collected from allogenic donated feces (Table 2). The recent approval of Rebyota as a new drug by the FDA was based on the allogenic healthy fecal matter.^[^ ^188^ ^]^ An intriguing idea proposed by Ke et al. suggests the creation of a personal microbial Noah's ark using stool banks to collect hosts' stool samples at optimal health and cryopreserve them for future autologous transplantation.^[^ ^189^ ^]^ This approach may increase the safety of transplanting bacteria or the virome in future practice. However, it is important to note that determining optimal health for stool collection requires further research, as environmental factors and diet can also significantly impact the gut microbiome in addition to host factors.^[^ ^190^, ^191^, ^192^ ^]^ Several studies involving at least 792 healthy adults in China have shown significant variation in virome, mycobiome, and archaeome between residents from rural and urban regions,^[^ ^190^, ^191^, ^192^ ^]^ with geography, urbanization, and diet have a strong impact on the microbiome.

## 7 Conclusions

Increasingly studies have shown that FMT has great potential in clinical practice for many diseases. The next generation of FMT for dysbiosis‐related diseases, including CDI, antibiotics‐associated dysbiosis, metabolic diseases, and others, will likely involve transplanting bacteria (including WMT and purified bacteria spores) or the virome (including FVT). While most current studies use allogenic donated feces for preparing transplants, autologous transplantation may also be an option in the future. All developed microbiota transplantation or microbiota‐based drugs can be delivered through colonic TET according to physicians’ decision. The new sequencing technology for providing genome‐length reads containing all mutations will help improve the development of FMT. In summary, the next generation of FMT is mainly moving forward for transplanting the enriched bacteria or the virome from healthy donors.

## Conflict of Interest

Faming Zhang conceived the concept of GenFMTer and transendoscopic enteral tubing and devices related to them. The remaining authors declare that the research was conducted in the absence of any commercial or financial relationships that could be construed as a potential conflict of interest.

## Author Contributions

Y.Y. conducted information collection and wrote the manuscript. W.W. revised the manuscript. F.Z. conceived the concept, revised the manuscript, and provided valuable suggestions for this study. All authors have read and agreed to the published version of the manuscript.

## References

[1] L. K. Ursell , J. L. Metcalf , L. W. Parfrey , R. Knight , Nutr Rev 2012, 70, S38. 22861806 10.1111/j.1753-4887.2012.00493.x PMC3426293

[2] M. L. Richard , H. Sokol , Nat. Rev. Gastroenterol. Hepatol. 2019, 16, 331. 30824884 10.1038/s41575-019-0121-2

[3] S. V. Lynch , O. Pedersen , N. Engl. J. Med. 2016, 375, 2369. 27974040 10.1056/NEJMra1600266

[4] J. R. Allegretti , B. H. Mullish , C. Kelly , M. Fischer , Lancet 2019, 394, 420. 31379333 10.1016/S0140-6736(19)31266-8

[5] F. Zhang , B. Cui , X. He , Y. Nie , K. Wu , D. Fan , Protein Cell 2018, 9, 462. 29691757 10.1007/s13238-018-0541-8 PMC5960466

[6] H. H. Choi , Y. S. Cho , Clin Endosc 2016, 49, 257. 26956193 10.5946/ce.2015.117 PMC4895930

[7] Y. Wang , S. Zhang , T. J. Borody , F. Zhang , Chin Med J (Engl) 2022, 135, 1927. 36103991 10.1097/CM9.0000000000002339 PMC9746749

[8] M. N. Quraishi , M. Widlak , N. Bhala , D. Moore , M. Price , N. Sharma , T. H. Iqbal , Aliment. Pharmacol. Ther. 2017, 46, 479. 28707337 10.1111/apt.14201

[9] D. P. Bojanova , S. R. Bordenstein , PLoS Biol. 2016, 14, 1002503. 10.1371/journal.pbio.1002503 PMC4942072 27404502

[10] R. Sender , S. Fuchs , R. Milo , PLoS Biol. 2016, 14, 1002533. 10.1371/journal.pbio.1002533 PMC4991899 27541692

[11] M. De Paepe , M. Leclerc , C. R. Tinsley , M. A. Petit , Front Cell Infect Microbiol 2014, 4, 39. 24734220 10.3389/fcimb.2014.00039 PMC3975094

[12] H. Duboc , S. Rajca , D. Rainteau , D. Benarous , M. A. Maubert , E. Quervain , G. Thomas , V. Barbu , L. Humbert , G. Despras , C. Bridonneau , F. Dumetz , J. P. Grill , J. Masliah , L. Beaugerie , J. Cosnes , O. Chazouilleres , R. Poupon , C. Wolf , J. M. Mallet , P. Langella , G. Trugnan , H. Sokol , P. Seksik , Gut 2013, 62, 531. 22993202 10.1136/gutjnl-2012-302578

[13] D. J. Morrison , T. Preston , Gut Microbes 2016, 7, 189. 26963409 10.1080/19490976.2015.1134082 PMC4939913

[14] T. S. B. Schmidt , S. S. Li , O. M. Maistrenko , W. Akanni , L. P. Coelho , S. Dolai , A. Fullam , A. M. Glazek , R. Hercog , H. Herrema , F. Jung , S. Kandels , A. Orakov , R. Thielemann , M. Von Stetten , T. Van Rossum , V. Benes , T. J. Borody , W. M. De Vos , C. Y. Ponsioen , M. Nieuwdorp , P. Bork , Nat. Med. 2022, 28, 1902. 36109636 10.1038/s41591-022-01913-0 PMC9499871

[15] S. P. Costello , W. Soo , R. V. Bryant , V. Jairath , A. L. Hart , J. M. Andrews , Aliment. Pharmacol. Ther. 2017, 46, 213. 28612983 10.1111/apt.14173

[16] Y. Xiao , M. T. Angulo , S. Lao , S. T. Weiss , Y.‐Y. Liu , Nat. Commun. 2020, 11, 3329. 32620839 10.1038/s41467-020-17180-x PMC7334230

[17] K. Fujimoto , Y. Kimura , J. R. Allegretti , M. Yamamoto , Y. Z. Zhang , K. Katayama , G. Tremmel , Y. Kawaguchi , M. Shimohigoshi , T. Hayashi , M. Uematsu , K. Yamaguchi , Y. Furukawa , Y. Akiyama , R. Yamaguchi , S. E. Crowe , P. B. Ernst , S. Miyano , H. Kiyono , S. Imoto , S. Uematsu , Gastroenterology 2021, 160, 2089. 33577875 10.1053/j.gastro.2021.02.013 PMC8684800

[18] G. Lu , W. Wang , P. Li , Q. Wen , B. Cui , F. Zhang , Microb Biotechnol 2022, 15, 2439. 35576458 10.1111/1751-7915.14074 PMC9437882

[19] S. J. Ott , G. H. Waetzig , A. Rehman , J. Moltzau‐Anderson , R. Bharti , J. A. Grasis , L. Cassidy , A. Tholey , H. Fickenscher , D. Seegert , P. Rosenstiel , S. Schreiber , Gastroenterology 2017, 152, 799. 27866880 10.1053/j.gastro.2016.11.010

[20] T. S. Rasmussen , C. M. J. Mentzel , W. Kot , J. L. Castro‐Mejía , S. Zuffa , J. R. Swann , L. H. Hansen , F. K. Vogensen , A. K. Hansen , D. S. Nielsen , Gut 2020, 69, 2122. 32165408 10.1136/gutjnl-2019-320005

[21] P. Feuerstadt , T. J. Louie , B. Lashner , E. E. L. Wang , L. Diao , J. A. Bryant , M. Sims , C. S. Kraft , S. H. Cohen , C. S. Berenson , L. Y. Korman , C. B. Ford , K. D. Litcofsky , M. J. Lombardo , J. R. Wortman , H. Wu , J. G. Aunins , C. W. J. Mcchalicher , J. A. Winkler , B. H. Mcgovern , M. Trucksis , M. R. Henn , L. Von Moltke , N. Engl. J. Med. 2022, 386, 220. 35045228 10.1056/NEJMoa2106516

[22] S. Lam , X. Bai , A. N. Shkoporov , H. Park , X. Wu , P. Lan , T. Zuo , Lancet Gastroenterol Hepatol 2022, 7, 472. 35276080 10.1016/S2468-1253(21)00303-4

[23] Y. Wang , B. Cui , F. Zhang , Curr Med Res Opin 2022, 38, 531. 35040380 10.1080/03007995.2022.2030563

[24] H. Wang , B. Cui , Q. Li , X. Ding , P. Li , T. Zhang , X. Yang , G. Ji , F. Zhang , Adv. Ther. 2018, 35, 1935. 30328062 10.1007/s12325-018-0800-3 PMC6223988

[25] B. H. McGovern , C. B. Ford , M. R. Henn , D. S. Pardi , S. Khanna , E. L. Hohmann , E. J. O'Brien , C. A. Desjardins , P. Bernardo , J. R. Wortman , M. J. Lombardo , K. D. Litcofsky , J. A. Winkler , C. W. J. McChalicher , S. S. Li , A. D. Tomlinson , M. Nandakumar , D. N. Cook , R. J. Pomerantz , J. G. Aunins , M. Trucksis , Clin. Infect. Dis. 2021, 72, 2132. 32255488 10.1093/cid/ciaa387 PMC8204772

[26] T. Zhang , G. Lu , Z. Zhao , Y. Liu , Q. Shen , P. Li , Y. Chen , H. Yin , H. Wang , C. Marcella , B. Cui , L. Cheng , G. Ji , F. Zhang , Protein Cell 2020, 11, 251. 31919742 10.1007/s13238-019-00684-8 PMC7093410

[27] Fecal Microbiota Transplantation‐standardization Study Group , Chin Med J (Engl) 2020, 133, 2330.32701590

[28] A. Howe , D. L. Ringus , R. J. Williams , Z. N. Choo , S. M. Greenwald , S. M. Owens , M. L. Coleman , F. Meyer , E. B. Chang , ISME J 2016, 10, 1217. 26473721 10.1038/ismej.2015.183 PMC5029215

[29] J. L. Moreno‐Gallego , S. P. Chou , S. C. Di Rienzi , J. K. Goodrich , T. D. Spector , J. T. Bell , N. D. Youngblut , I. Hewson , A. Reyes , R. E. Ley , Cell Host Microbe 2019, 25, 261. 30763537 10.1016/j.chom.2019.01.019 PMC6411085

[30] J. Guilfoyle , J. Considine , S. L. Bouchoucha , J Clin Nurs 2021, 30, 1236. 33377562 10.1111/jocn.15625

[31] B. Mcsweeney , J. R. Allegretti , M. Fischer , H. Xu , K. J. Goodman , T. Monaghan , C. Mcleod , B. H. Mullish , E. O. Petrof , E. L. Phelps , R. Chis , A. Edmison , A. Juby , R. Ennis‐Davis , B. Roach , K. Wong , D. Kao , Gut Microbes 2020, 11, 51. 31122134 10.1080/19490976.2019.1611153 PMC6973337

[32] A. G. Al‐Bakri , A. A. Akour , W. K. Al‐Delaimy , BMC Med. Ethics 2021, 22, 19. 33639935 10.1186/s12910-021-00587-6 PMC7912465

[33] A. Reyes , M. Haynes , N. Hanson , F. E. Angly , A. C. Heath , F. Rohwer , J. I. Gordon , Nature 2010, 466, 334. 20631792 10.1038/nature09199 PMC2919852

[34] M. Dalmasso , C. Hill , R. P. Ross , Trends Microbiol. 2014, 22, 399. 24656964 10.1016/j.tim.2014.02.010

[35] T. S. Rasmussen , A. K. Koefoed , R. R. Jakobsen , L. Deng , J. L. Castro‐Mejía , A. Brunse , H. Neve , F. K. Vogensen , D. S. Nielsen , FEMS Microbiol. Rev. 2020, 44, 507. 32495834 10.1093/femsre/fuaa020

[36] M. Dsouza , R. Menon , E. Crossette , S. K. Bhattarai , J. Schneider , Y.‐G. Kim , S. Reddy , S. Caballero , C. Felix , L. Cornacchione , J. Hendrickson , A. R. Watson , S. S. Minot , N. Greenfield , L. Schopf , R. Szabady , J. Patarroyo , W. Smith , P. Harrison , E. J. Kuijper , C. P. Kelly , B. Olle , D. Bobilev , J. L. Silber , V. Bucci , B. Roberts , J. Faith , J. M. Norman , Cell Host Microbe 2022, 30, 583. 35421353 10.1016/j.chom.2022.03.016

[37] M. Titécat , C. Rousseaux , C. Dubuquoy , B. Foligné , O. Rahmouni , S. Mahieux , P. Desreumaux , J. Woolston , A. Sulakvelidze , K. Wannerberger , C. Neut , J Crohns Colitis 2022, 16, 1617. 35997152 10.1093/ecco-jcc/jjac064

[38] S. Federici , S. Kredo‐Russo , R. Valdés‐Mas , D. Kviatcovsky , E. Weinstock , Y. Matiuhin , Y. Silberberg , K. Atarashi , M. Furuichi , A. Oka , B. Liu , M. Fibelman , I. N. Weiner , E. Khabra , N. Cullin , N. Ben‐Yishai , D. Inbar , H. Ben‐David , J. Nicenboim , N. Kowalsman , W. Lieb , E. Kario , T. Cohen , Y. F. Geffen , L. Zelcbuch , A. Cohen , U. Rappo , I. Gahali‐Sass , M. Golembo , V. Lev , et al., Cell 2022, 185, 2879. 35931020 10.1016/j.cell.2022.07.003

[39] F. Zhang , W. Wang , Y. Nie , J. Li , X. He , Microb Biotechnol 2023, 16, 1705. 37452703 10.1111/1751-7915.14317 PMC10443319

[40] B. Eiseman , W. Silen , G. S. Bascom , A. J. Kauvar , Surgery 1958, 44, 854. 13592638

[41] A. Schwan , Lancet 1983, 2, 845. 10.1016/s0140-6736(83)90753-5 6137662

[42] C. M. Surawicz , L. J. Brandt , D. G. Binion , A. N. Ananthakrishnan , S. R. Curry , P. H. Gilligan , L. V. Mcfarland , M. Mellow , B. S. Zuckerbraun , Am. J. Gastroenterol. 2013, 108, 478. 23439232 10.1038/ajg.2013.4

[43] F. Zhang , P. Yang , Y. Chen , R. Wang , B. Liu , J. Wang , M. Yuan , L. Zhang , Front Cell Infect Microbiol 2022, 12, 1057492. 36439220 10.3389/fcimb.2022.1057492 PMC9684174

[44] D. W. Kang , J. B. Adams , A. C. Gregory , T. Borody , L. Chittick , A. Fasano , A. Khoruts , E. Geis , J. Maldonado , S. McDonough‐Means , E. L. Pollard , S. Roux , M. J. Sadowsky , K. S. Lipson , M. B. Sullivan , J. G. Caporaso , R. Krajmalnik‐Brown , Microbiome 2017, 5, 10. 28122648 10.1186/s40168-016-0225-7 PMC5264285

[45] P. Zheng , B. Zeng , C. Zhou , M. Liu , Z. Fang , X. Xu , L. Zeng , J. Chen , S. Fan , X. Du , X. Zhang , D. Yang , Y. Yang , H. Meng , W. Li , N. D. Melgiri , J. Licinio , H. Wei , P. Xie , Mol. Psychiatry 2016, 21, 786. 27067014 10.1038/mp.2016.44

[46] X. F. Hu , W. Y. Zhang , Q. Wen , W. J. Chen , Z. M. Wang , J. Chen , F. Zhu , K. Liu , L. X. Cheng , J. Yang , Y. W. Shu , Pharmacol Res 2019, 139, 412. 30508676 10.1016/j.phrs.2018.11.042

[47] X. Yan , J. Jin , X. Su , X. Yin , J. Gao , X. Wang , S. Zhang , P. Bu , M. Wang , Y. Zhang , Z. Wang , Q. Zhang , Circ. Res. 2020, 126, 839. 32078445 10.1161/CIRCRESAHA.119.316394

[48] T. J. Schuijt , J. M. Lankelma , B. P. Scicluna , F. De Sousa E Melo , J. J. T. H. Roelofs , J. D. De Boer , A. J. Hoogendijk , R. De Beer , A. De Vos , C. Belzer , W. M. De Vos , T. Van Der Poll , W. J. Wiersinga , Gut 2016, 65, 575. 26511795 10.1136/gutjnl-2015-309728 PMC4819612

[49] J. J. Witjes , L. P. Smits , C. T. Pekmez , A. Prodan , A. S. Meijnikman , M. A. Troelstra , K. E. C. Bouter , H. Herrema , E. Levin , A. G. Holleboom , M. Winkelmeijer , U. H. Beuers , K. van Lienden , J. Aron‐Wisnewky , V. Mannisto , J. J. Bergman , J. H. Runge , A. J. Nederveen , L. O. Dragsted , P. Konstanti , E. G. Zoetendal , W. de Vos , J. Verheij , A. K. Groen , M. Nieuwdorp , Hepatol Commun 2020, 4, 1578. 33163830 10.1002/hep4.1601 PMC7603524

[50] L. Craven , A. Rahman , S. Nair Parvathy , M. Beaton , J. Silverman , K. Qumosani , I. Hramiak , R. Hegele , T. Joy , J. Meddings , B. Urquhart , R. Harvie , C. Mckenzie , K. Summers , G. Reid , J. P. Burton , M. Silverman , Am. J. Gastroenterol. 2020, 115, 1055. 32618656 10.14309/ajg.0000000000000661

[51] X. Qi , X. Li , Y. Zhao , X. Wu , F. Chen , X. Ma , F. Zhang , D. Wu , Front Immunol 2018, 9, 2195. 30319644 10.3389/fimmu.2018.02195 PMC6167440

[52] C. Huang , P. Yi , M. Zhu , W. Zhou , B. Zhang , X. Yi , H. Long , G. Zhang , H. Wu , G. C. Tsokos , M. Zhao , Q. Lu , J Autoimmun 2022, 130, 102844. 35690527 10.1016/j.jaut.2022.102844

[53] J. H. Kim , K. Kim , W. Kim , Exp. Mol. Med. 2021, 53, 907. 34017060 10.1038/s12276-021-00627-6 PMC8178377

[54] W. Wang , G. Lu , X. Wu , Q. Wen , F. Zhang , J Clin Med 2023, 12, 780. 36769429 10.3390/jcm12030780 PMC9918197

[55] X. Wu , R. Ai , J. Xu , Q. Wen , H. Pan , Z. Zhang , N. Wang , Y. Fang , D. Ding , Q. Wang , S. Han , X. Liu , M. Wu , Z. Jia , J. Song , T. Lin , B. Cui , Y. Nie , X. Wang , F. Zhang , J. Dig. Dis. 2023. 10.1111/1751-2980.13227 37681235

[56] X. Ding , Q. Li , P. Li , T. Zhang , B. Cui , G. Ji , X. Lu , F. Zhang , Drug Saf. 2019, 42, 869. 30972640 10.1007/s40264-019-00809-2

[57] A. N. Shkoporov , S. R. Stockdale , A. Lavelle , I. Kondova , C. Heuston , A. Upadrasta , E. V. Khokhlova , I. van der Kamp , B. Ouwerling , L. A. Draper , J. A. M. Langermans , R. Paul Ross , C. Hill , Nat. Microbiol. 2022, 7, 1301. 35918425 10.1038/s41564-022-01178-w PMC7614033

[58] D. Shalon , R. N. Culver , J. A. Grembi , J. Folz , P. V. Treit , H. Shi , F. A. Rosenberger , L. Dethlefsen , X. Meng , E. Yaffe , A. Aranda‐Díaz , P. E. Geyer , J. B. Mueller‐Reif , S. Spencer , A. D. Patterson , G. Triadafilopoulos , S. P. Holmes , M. Mann , O. Fiehn , D. A. Relman , K. C. Huang , Nature 2023, 617, 581. 37165188 10.1038/s41586-023-05989-7 PMC10191855

[59] S. Khanna , M. Assi , C. Lee , D. Yoho , T. Louie , W. Knapple , H. Aguilar , J. Garcia‐Diaz , G. P. Wang , S. M. Berry , J. Marion , X. Su , T. Braun , L. Bancke , P. Feuerstadt , Drugs 2022, 82, 1527. 36287379 10.1007/s40265-022-01797-x PMC9607700

[60] C. Marcella , B. Cui , C. R. Kelly , G. Ianiro , G. Cammarota , F. Zhang , Aliment. Pharmacol. Ther. 2021, 53, 33. 33159374 10.1111/apt.16148

[61] M. Baxter , T. Ahmad , A. Colville , R. Sheridan , Clin. Infect. Dis. 2015, 61, 136. 25805303 10.1093/cid/civ247

[62] N. A. Cohen , D. M. Livovsky , S. Yaakobovitch , M. Ben Yehoyada , R. Ben Ami , A. Adler , H. Guzner‐Gur , E. Goldin , M. E. Santo , Z. Halpern , K. Paz , N. Maharshak , Isr. Med. Assoc. J. 2016, 18, 594. 28471618

[63] Y. H. Van Beurden , P. F. De Groot , E. Van Nood , M. Nieuwdorp , J. J. Keller , A. Goorhuis , United European Gastroenterol J 2017, 5, 868. 10.1177/2050640616678099 PMC5625865 29026601

[64] C. R. Kelly , C. Ihunnah , M. Fischer , A. Khoruts , C. Surawicz , A. Afzali , O. Aroniadis , A. Barto , T. Borody , A. Giovanelli , S. Gordon , M. Gluck , E. L. Hohmann , D. Kao , J. Y. Kao , D. P. Mcquillen , M. Mellow , K. M. Rank , K. Rao , A. Ray , M. A. Schwartz , N. Singh , N. Stollman , D. L. Suskind , S. M. Vindigni , I. Youngster , L. Brandt , Am. J. Gastroenterol. 2014, 109, 1065. 24890442 10.1038/ajg.2014.133 PMC5537742

[65] Z. Defilipp , P. P. Bloom , M. Torres Soto , M. K. Mansour , M. R. A. Sater , M. H. Huntley , S. Turbett , R. T. Chung , Y.‐B. Chen , E. L. Hohmann , N. Engl. J. Med. 2019, 381, 2043. 31665575 10.1056/NEJMoa1910437

[66] T. G. Gweon , Y. J. Lee , K. O. Kim , S. K. Yim , J. S. Soh , S. Y. Kim , J. J. Park , S. Y. Shin , T. H. Lee , C. H. Choi , Y. S. Cho , D. Yong , J. W. Chung , K. J. Lee , O. Y. Lee , M. G. Choi , M. Choi , M. Gut , J Neurogastroenterol Motil 2022, 28, 28. 34980687 10.5056/jnm21221 PMC8748844

[67] C. Haifer , C. R. Kelly , S. Paramsothy , D. Andresen , L. E. Papanicolas , G. L. Mckew , T. J. Borody , M. Kamm , S. P. Costello , J. M. Andrews , J. Begun , H. T. Chan , S. Connor , S. Ghaly , P. D. Johnson , D. A. Lemberg , R. Paramsothy , A. Redmond , H. Sheorey , D. Van Der Poorten , R. W. Leong , Gut 2020, 69, 801. 32047093 10.1136/gutjnl-2019-320260

[68] B. H. Mullish , M. N. Quraishi , J. P. Segal , V. L. Mccune , M. Baxter , G. L. Marsden , D. J. Moore , A. Colville , N. Bhala , T. H. Iqbal , C. Settle , G. Kontkowski , A. L. Hart , P. M. Hawkey , S. D. Goldenberg , H. R. T. Williams , Gut 2018, 67, 1920. 30154172 10.1136/gutjnl-2018-316818

[69] M. Osman , S. Budree , C. R. Kelly , P. Panchal , J. R. Allegretti , Z. Kassam , S. W. Olesen , B. Ramakrishna , N. Dubois , K. O'Brien , M. Fischer , N. Stollman , R. A. Hays , C. P. Kelly , K. Amaratunga , T. Qazi , J. W. Crothers , A. Abend , M. Bougas , L. Burns , I. Decaille‐Hodge , M. Dickens , C. Edelstein , D. Gabdrakhmanova , C. Kerwin , R. Landry , K. Ling , D. Martin , G. Medina , G. Mendolia , et al., Gastroenterology 2022, 163, 319. 35398345 10.1053/j.gastro.2022.03.051

[70] M. P. Spindler , S. Siu , I. Mogno , Z. Li , C. Yang , S. Mehandru , G. J. Britton , J. J. Faith , Cell Host Microbe 2022, 30, 1481. 36099923 10.1016/j.chom.2022.08.009 PMC9588646

[71] S. C. Ng , M. A. Kamm , Y. K. Yeoh , P. K. S. Chan , T. Zuo , W. Tang , A. Sood , A. Andoh , N. Ohmiya , Y. Zhou , C. J. Ooi , V. Mahachai , C. Y. Wu , F. Zhang , K. Sugano , F. K. L. Chan , Gut 2020, 69, 83. 31611298 10.1136/gutjnl-2019-319407 PMC6943253

[72] G. Cammarota , G. Ianiro , C. R. Kelly , B. H. Mullish , J. R. Allegretti , Z. Kassam , L. Putignani , M. Fischer , J. J. Keller , S. P. Costello , H. Sokol , P. Kump , R. Satokari , S. A. Kahn , D. Kao , P. Arkkila , E. J. Kuijper , M. J. G. Vehreschild , C. Pintus , L. Lopetuso , L. Masucci , F. Scaldaferri , E. M. Terveer , M. Nieuwdorp , A. Lopez‐Sanroman , J. Kupcinskas , A. Hart , H. Tilg , A. Gasbarrini , Gut 2019, 68, 2111. 31563878 10.1136/gutjnl-2019-319548 PMC6872442

[73] G. Cammarota , G. Ianiro , H. Tilg , M. Rajilic‐Stojanovic , P. Kump , R. Satokari , H. Sokol , P. Arkkila , C. Pintus , A. Hart , J. Segal , M. Aloi , L. Masucci , A. Molinaro , F. Scaldaferri , G. Gasbarrini , A. Lopez‐Sanroman , A. Link , P. De Groot , W. M. De Vos , C. Högenauer , P. Malfertheiner , E. Mattila , T. Milosavljevic , M. Nieuwdorp , M. Sanguinetti , M. Simren , A. Gasbarrini , Gut 2017, 66, 569. 28087657 10.1136/gutjnl-2016-313017 PMC5529972

[74] S. Dogra , C. Oneto , A. Sherman , R. Varughese , A. Yuen , I. Sherman , A. Cohen , Y. Luo , L. A. Chen , J Clin Gastroenterol 2022. 10.1097/MCG.0000000000001778 PMC10102254 36227005

[75] J. Zhang , Y. Guo , L. Duan , Front Med (Lausanne) 2022, 9, 773105. 35721102 10.3389/fmed.2022.773105 PMC9198717

[76] Y. Ma , J. Liu , C. Rhodes , Y. Nie , F. Zhang , Am J Bioeth 2017, 17, 34. 10.1080/15265161.2017.1299240 28430065

[77] M. Mikail , K. C. O'doherty , S. M. Poutanen , S. S. Hota , Lancet Infect. Dis. 2020, 20, e44. 31784367 10.1016/S1473-3099(19)30569-9

[78] Z. Grigoryan , M. J. Shen , S. W. Twardus , M. M. Beuttler , L. A. Chen , A. Bateman‐House , Med Microecol 2020, 6, 100027. 33834162 10.1016/j.medmic.2020.100027 PMC8026161

[79] A. Scheeler , Medicine & Ethics 2021, 47, 524. 10.1177/1073110519897729 31957572

[80] E. M. Terveer , Y. H. Van Beurden , A. Goorhuis , J. F. M. L. Seegers , M. P. Bauer , E. Van Nood , M. G. W. Dijkgraaf , C. J. J. Mulder , C. M. J. E. Vandenbroucke‐Grauls , H. W. Verspaget , J. J. Keller , E. J. Kuijper , Clin Microbiol. Infect. 2017, 23, 924. 28529025 10.1016/j.cmi.2017.05.015

[81] H. L. Huang , H. T. Chen , Q. L. Luo , H. M. Xu , J. He , Y. Q. Li , Y. L. Zhou , F. Yao , Y. Q. Nie , Y. J. Zhou , J Dig Dis 2019, 20, 401. 31070838 10.1111/1751-2980.12756

[82] M. Biazzo , G. Deidda , J Clin Med 2022, 11, 4119. 35887883 10.3390/jcm11144119 PMC9320118

[83] J. W. Wang , Y. K. Wang , F. Zhang , Y.‐C. Su , J. Y. Wang , D. C. Wu , W. H. Hsu , Kaohsiung J Med Sci 2019, 35, 566. 31197926 10.1002/kjm2.12094 PMC11900732

[84] M. Gulati , S. K. Singh , L. Corrie , I. P. Kaur , L. Chandwani , Pharmacol Res 2020, 159, 104954. 32492490 10.1016/j.phrs.2020.104954

[85] X. Liu , M. Dai , Y. Ma , N. Zhao , Z. Wang , Y. Yu , Y. Xu , H. Zhang , L. Xiang , H. Tian , G. Shui , F. Zhang , J. Wang , Engineering 2022, 15, 89.

[86] S. Khanna , D. S. Pardi , C. R. Kelly , C. S. Kraft , T. Dhere , M. R. Henn , M. J. Lombardo , M. Vulic , T. Ohsumi , J. Winkler , C. Pindar , B. H. Mcgovern , R. J. Pomerantz , J. G. Aunins , D. N. Cook , E. L. Hohmann , J Infect Dis 2016, 214, 173. 26908752 10.1093/infdis/jiv766

[87] I. Leonardi , S. Paramsothy , I. Doron , A. Semon , N. O. Kaakoush , J. C. Clemente , J. J. Faith , T. J. Borody , H. M. Mitchell , J. F. Colombel , M. A. Kamm , I. D. Iliev , Cell Host Microbe 2020, 27, 823. 32298656 10.1016/j.chom.2020.03.006 PMC8647676

[88] X. Wu , B.‐T. Cui , F.‐M. Zhang , Chin Med J (Engl) 2021, 134, 741. 33470649 10.1097/CM9.0000000000001212 PMC7990002

[89] L. Wu , M. Q. Li , Y.‐T. Xie , Q. Zhang , X. J. Lu , T. Liu , W. Y. Lin , J. T. Xu , Q. P. Wu , X. X. He , Front Endocrinol (Lausanne) 2022, 13, 985636. 36213281 10.3389/fendo.2022.985636 PMC9539914

[90] J. Yang , X. Yang , G. Wu , F. Huang , X. Shi , W. Wei , Y. Zhang , H. Zhang , L. Cheng , L. Yu , J. Shang , Y. Lv , X. Wang , R. Zhai , P. Li , B. Cui , Y. Fang , X. Deng , S. Tang , L. Wang , Q. Yuan , L. Zhao , F. Zhang , C. Zhang , H. Yuan , Cell Metab. 2023, 35, 1548. 37451270 10.1016/j.cmet.2023.06.010

[91] W. He , O. Rebello , R. Savino , R. Terracciano , C. Schuster‐Klein , B. Guardiola , K. Maedler , Biochim Biophys Acta Mol Basis Dis 2019, 1865, 86. 30287405 10.1016/j.bbadis.2018.09.030

[92] F. Z. Marques , C. R. Mackay , D. M. Kaye , Nat Rev Cardiol 2018, 15, 20. 28836619 10.1038/nrcardio.2017.120

[93] T. Yang , E. M. Richards , C. J. Pepine , M. K. Raizada , Nat Rev Nephrol 2018, 14, 442. 29760448 10.1038/s41581-018-0018-2 PMC6385605

[94] T. Yang , M. M. Santisteban , V. Rodriguez , E. Li , N. Ahmari , J. M. Carvajal , M. Zadeh , M. Gong , Y. Qi , J. Zubcevic , B. Sahay , C. J. Pepine , M. K. Raizada , M. Mohamadzadeh , Hypertension 2015, 65, 1331. 25870193 10.1161/HYPERTENSIONAHA.115.05315 PMC4433416

[95] M. Toral , I. Robles‐Vera , N. De La Visitación , M. Romero , T. Yang , M. Sánchez , M. Gómez‐Guzmán , R. Jiménez , M. K. Raizada , J. Duarte , Front Physiol 2019, 10, 231. 30930793 10.3389/fphys.2019.00231 PMC6423906

[96] H. J. Zhong , H. L. Zeng , Y.‐L. Cai , Y.‐P. Zhuang , Y.‐L. Liou , Q. Wu , X. X. He , Front Cell Infect Microbiol 2021, 11, 679624. 34458158 10.3389/fcimb.2021.679624 PMC8385408

[97] B. T. Tierney , Y. Tan , A. D. Kostic , C. J. Patel , Nat. Commun. 2021, 12, 2907. 34006865 10.1038/s41467-021-23029-8 PMC8131609

[98] F. Liang , X. Lu , Z. Deng , H. J. Zhong , W. Zhang , Q. Li , H. H. Zhou , Y.‐L. Liou , X. X. He , Front Endocrinol (Lausanne) 2022, 13, 827107. 35528013 10.3389/fendo.2022.827107 PMC9074302

[99] G. Lu , Q. Wen , B. Cui , Q. Li , F. Zhang , J Biomed Res 2022, 37, 69. 35821195 10.7555/JBR.36.20220088 PMC9898040

[100] D.‐W. Kang , J. B. Adams , A. C. Gregory , T. Borody , L. Chittick , A. Fasano , A. Khoruts , E. Geis , J. Maldonado , S. McDonough‐Means , E. L. Pollard , S. Roux , M. J. Sadowsky , K. S. Lipson , M. B. Sullivan , J. G. Caporaso , R. Krajmalnik‐Brown , Microbiome 2017, 5, 10. 28122648 10.1186/s40168-016-0225-7 PMC5264285

[101] Z. Y. Pan , H. J. Zhong , D. N. Huang , L. H. Wu , X. X. He , Front Pediatr 2022, 10, 928785. 35783298 10.3389/fped.2022.928785 PMC9249087

[102] J. F. Cryan , T. G. Dinan , Nat. Rev. Neurosci. 2012, 13, 701. 22968153 10.1038/nrn3346

[103] M. Guo , J. Zhu , T. Yang , X. Lai , X. Liu , J. Liu , J. Chen , T. Li , Brain Res. Bull. 2018, 137, 35. 29122693 10.1016/j.brainresbull.2017.11.001

[104] M. Dai , Y. Liu , W. Chen , H. Buch , Y. Shan , L. Chang , Y. Bai , C. Shen , X. Zhang , Y. Huo , D. Huang , Z. Yang , Z. Hu , X. He , J. Pan , L. Hu , X. Pan , X. Wu , B. Deng , Z. Li , B. Cui , F. Zhang , Crit. Care 2019, 23, 324. 31639033 10.1186/s13054-019-2604-5 PMC6805332

[105] X. Ding , Q. Li , P. Li , X. Chen , L. Xiang , L. Bi , J. Zhu , X. Huang , B. Cui , F. Zhang , Radiother Oncol 2020, 143, 12. 32044171 10.1016/j.radonc.2020.01.011

[106] D. M. Lin , B. Koskella , N. L. Ritz , D. Lin , A. Carroll‐Portillo , H. C. Lin , Front Cell Infect Microbiol 2019, 9, 348. 31750259 10.3389/fcimb.2019.00348 PMC6843071

[107] L. A. Draper , F. J. Ryan , M. Dalmasso , P. G. Casey , A. Mccann , V. Velayudhan , R. P. Ross , C. Hill , BMC Biol. 2020, 18, 173. 33218339 10.1186/s12915-020-00906-0 PMC7679995

[108] A. Brunse , L. Deng , X. Pan , Y. Hui , J. L. Castro‐Mejia , W. Kot , D. N. Nguyen , J. B. Secher , D. S. Nielsen , T. Thymann , ISME J 2021, 16, 686. 34552194 10.1038/s41396-021-01107-5 PMC8857206

[109] C. M. Theriot , V. B. Young , Annu. Rev. Microbiol. 2015, 69, 445. 26488281 10.1146/annurev-micro-091014-104115 PMC4892173

[110] D. N. Gerding , T. Meyer , C. Lee , S. H. Cohen , U. K. Murthy , A. Poirier , T. C. Van Schooneveld , D. S. Pardi , A. Ramos , M. A. Barron , H. Chen , S. Villano , JAMA, J. Am. Med. Assoc. 2015, 313, 1719. 10.1001/jama.2015.3725 25942722

[111] S. H. Cohen , T. J. Louie , M. Sims , E. E. L. Wang , A. Memisoglu , B. H. Mcgovern , L. Von Moltke , JAMA, J. Am. Med. Assoc. 2022, 328, 2062. 10.1001/jama.2022.16476 PMC9582966 36260754

[112] A. Benítez‐Páez , A. V. Hartstra , M. Nieuwdorp , Y. Sanz , Gut Microbes 2022, 14, 2078621. 35604764 10.1080/19490976.2022.2078621 PMC9132484

[113] G. Ianiro , M. Punčochář , N. Karcher , S. Porcari , F. Armanini , F. Asnicar , F. Beghini , A. Blanco‐Míguez , F. Cumbo , P. Manghi , F. Pinto , L. Masucci , G. Quaranta , S. De Giorgi , G. D. Sciumè , S. Bibbò , F. Del Chierico , L. Putignani , M. Sanguinetti , A. Gasbarrini , M. Valles‐Colomer , G. Cammarota , N. Segata , Nat. Med. 2022, 28, 1913. 36109637 10.1038/s41591-022-01964-3 PMC9499858

[114] T. Louie , Y. Golan , S. Khanna , D. Bobilev , N. Erpelding , C. Fratazzi , M. Carini , R. Menon , M. Ruisi , J. M. Norman , J. J. Faith , B. Olle , M. Li , J. L. Silber , D. S. Pardi , JAMA, J. Am. Med. Assoc. 2023, 329, 1356. 10.1001/jama.2023.4314 PMC10105904 37060545

[115] T. M. Santiago‐Rodriguez , B. L. Francois , J. M. Macklaim , E. Doukhanine , E. B. Hollister , Microorganisms 2023, 11, 1222. 37317196 10.3390/microorganisms11051222 PMC10223452

[116] C. Haifer , L. D. W. Luu , S. Paramsothy , T. J. Borody , R. W. Leong , N. O. Kaakoush , Gut 2022. 10.1136/gutjnl-2022-327742 35879048

[117] D. G. Maghini , M. Dvorak , A. Dahlen , M. Roos , S. Kuersten , A. S. Bhatt , Nat. Biotechnol. 2023.

[118] F. W. Twort , Lancet 1915, 186, 1241.

[119] N. Chanishvili , Adv Virus Res 2012, 83, 3. 22748807 10.1016/B978-0-12-394438-2.00001-3

[120] S. Minot , A. Bryson , C. Chehoud , G. D. Wu , J. D. Lewis , F. D. Bushman , Proc Natl Acad Sci U S A 2013, 110, 12450. 23836644 10.1073/pnas.1300833110 PMC3725073

[121] T. Zuo , S. H. Wong , K. Lam , R. Lui , K. Cheung , W. Tang , J. Y. L. Ching , P. K. S. Chan , M. C. W. Chan , J. C. Y. Wu , F. K. L. Chan , J. Yu , J. J. Y. Sung , S. C. Ng , Gut 2018, 67, 634. 28539351 10.1136/gutjnl-2017-313952 PMC5868238

[122] A. G. Clooney , T. D. S. Sutton , A. N. Shkoporov , R. K. Holohan , K. M. Daly , O. O'Regan , F. J. Ryan , L. A. Draper , S. E. Plevy , R. P. Ross , C. Hill , Cell Host Microbe 2019, 26, 764. 31757768 10.1016/j.chom.2019.10.009

[123] J. M. Norman , S. A. Handley , M. T. Baldridge , L. Droit , C. Y. Liu , B. C. Keller , A. Kambal , C. L. Monaco , G. Zhao , P. Fleshner , T. S. Stappenbeck , D. P. B. Mcgovern , A. Keshavarzian , E. A. Mutlu , J. Sauk , D. Gevers , R. J. Xavier , D. Wang , M. Parkes , H. W. Virgin , Cell 2015, 160, 447. 25619688 10.1016/j.cell.2015.01.002 PMC4312520

[124] P. Manrique , Y. Zhu , J. Van Der Oost , H. Herrema , M. Nieuwdorp , W. M. De Vos , M. Young , Gut Microbes 2021, 13, 1897217. 33794724 10.1080/19490976.2021.1897217 PMC8023239

[125] F. Zhang , T. Zuo , Y. K. Yeoh , F. W. T. Cheng , Q. Liu , W. Tang , K. C. Y. Cheung , K. Yang , C. P. Cheung , C. C. Mo , M. Hui , F. K. L. Chan , C.‐K. Li , P. K. S. Chan , S. C. Ng , Nat. Commun. 2021, 12, 65. 33397897 10.1038/s41467-020-20240-x PMC7782528

[126] F. Chen , S. Li , R. Guo , F. Song , Y. Zhang , X. Wang , X. Huo , Q. Lv , H. Ullah , G. Wang , Y. Ma , Q. Yan , X. Ma , J Adv Res 2022, 49, 103. 36198381 10.1016/j.jare.2022.09.012 PMC10334131

[127] L. A. Draper , F. J. Ryan , M. K. Smith , J. Jalanka , E. Mattila , P. A. Arkkila , R. P. Ross , R. Satokari , C. Hill , Microbiome 2018, 6, 220. 30526683 10.1186/s40168-018-0598-x PMC6288847

[128] F. Broecker , G. Russo , J. Klumpp , K. Moelling , Gut Microbes 2017, 8, 214. 27935413 10.1080/19490976.2016.1265196 PMC5479397

[129] D. Sheehan , C. Moran , F. Shanahan , J Gastroenterol 2015, 50, 495. 25808229 10.1007/s00535-015-1064-1

[130] I. D. Iliev , K. Cadwell , Gastroenterology 2021, 160, 1050. 33347881 10.1053/j.gastro.2020.06.100 PMC7956156

[131] A. Sinha , Y. Li , M. K. Mirzaei , M. Shamash , R. Samadfam , I. L. King , C. F. Maurice , Microbiome 2022, 10, 105. 35799219 10.1186/s40168-022-01275-2 PMC9264660

[132] S. Minot , R. Sinha , J. Chen , H. Li , S. A. Keilbaugh , G. D. Wu , J. D. Lewis , F. D. Bushman , Genome Res. 2011, 21, 1616. 21880779 10.1101/gr.122705.111 PMC3202279

[133] S. R. Modi , H. H. Lee , C. S. Spina , J. J. Collins , Nature 2013, 499, 219. 23748443 10.1038/nature12212 PMC3710538

[134] Y. Shono , M. R. M. Van Den Brink , Nat. Rev. Cancer 2018, 18, 283. 29449660 10.1038/nrc.2018.10 PMC7485905

[135] J. U. Peled , A. L. C. Gomes , S. M. Devlin , E. R. Littmann , Y. Taur , A. D. Sung , D. Weber , D. Hashimoto , A. E. Slingerland , J. B. Slingerland , M. Maloy , A. G. Clurman , C. K. Stein‐Thoeringer , K. A. Markey , M. D. Docampo , M. Burgos da Silva , N. Khan , A. Gessner , J. A. Messina , K. Romero , M. V. Lew , A. Bush , L. Bohannon , D. G. Brereton , E. Fontana , L. A. Amoretti , R. J. Wright , G. K. Armijo , Y. Shono , M. Sanchez‐Escamilla , et al., N. Engl. J. Med. 2020, 382, 822. 32101664 10.1056/NEJMoa1900623 PMC7534690

[136] L. Feghoul , S. Chevret , A. Cuinet , J.‐H. Dalle , M. Ouachée , K. Yacouben , M. Fahd , V. Guérin‐El Khourouj , J. Roupret‐Serzec , G. Sterkers , A. Baruchel , F. Simon , J. Legoff , Clin Microbiol. Infect. 2015, 21, 701. 25882354 10.1016/j.cmi.2015.03.011

[137] C. Pichereau , K. Desseaux , A. Janin , C. Scieux , R. Peffault De Latour , A. Xhaard , M. Robin , P. Ribaud , F. Agbalika , S. Chevret , G. Socié , Biol Blood Marrow Transplant 2012, 18, 141. 21801705 10.1016/j.bbmt.2011.07.018

[138] D. M. Zerr , M. Boeckh , C. Delaney , P. J. Martin , H. Xie , A. L. Adler , M.‐L. Huang , L. Corey , W. M. Leisenring , Biol Blood Marrow Transplant 2012, 18, 1700. 22641196 10.1016/j.bbmt.2012.05.012 PMC3439599

[139] J. R. Wingard , N. S. Majhail , R. Brazauskas , Z. Wang , K. A. Sobocinski , D. Jacobsohn , M. L. Sorror , M. M. Horowitz , B. Bolwell , J. D. Rizzo , G. Socié , J. Clin. Oncol. 2011, 29, 2230. 21464398 10.1200/JCO.2010.33.7212 PMC3107742

[140] P. Wohlfarth , M. Leiner , C. Schoergenhofer , G. Hopfinger , I. Goerzer , E. Puchhammer‐Stoeckl , W. Rabitsch , Biol. Blood Marr. Transpl. 2018, 24, 194. 10.1016/j.bbmt.2017.09.020 29032273

[141] J. Legoff , M. Resche‐Rigon , J. Bouquet , M. Robin , S. N. Naccache , S. Mercier‐Delarue , S. Federman , E. Samayoa , C. Rousseau , P. Piron , N. Kapel , F. Simon , G. Socié , C. Y. Chiu , Nat. Med. 2017, 23, 1080. 28759053 10.1038/nm.4380

[142] Y. Yang , L. Du , D. Shi , C. Kong , J. Liu , G. Liu , X. Li , Y. Ma , Nat. Commun. 2021, 12, 6757. 34799562 10.1038/s41467-021-27112-y PMC8604900

[143] E. Kernbauer , Y. Ding , K. Cadwell , Nature 2014, 516, 94. 25409145 10.1038/nature13960 PMC4257755

[144] M. Pammi , J. Cope , P. I. Tarr , B. B. Warner , A. L. Morrow , V. Mai , K. E. Gregory , J. S. Kroll , V. McMurtry , M. J. Ferris , L. Engstrand , H. E. Lilja , E. B. Hollister , J. Versalovic , J. Neu , Microbiome 2017, 5, 31. 28274256 10.1186/s40168-017-0248-8 PMC5343300

[145] S. N. Rajpathak , M. J. Gunter , J. Wylie‐Rosett , G. Y. F. Ho , R. C. Kaplan , R. Muzumdar , T. E. Rohan , H. D. Strickler , Diabetes Metab Res Rev 2009, 25, 3. 19145587 10.1002/dmrr.919 PMC4153414

[146] R. Kruse , S. G. Vienberg , B. F. Vind , B. Andersen , K. Højlund , Diabetologia 2017, 60, 2042. 28721439 10.1007/s00125-017-4373-5

[147] B. Dubern , K. Clement , Biochimie 2012, 94, 2111. 22627381 10.1016/j.biochi.2012.05.010

[148] A. E. Charos , B. D. Reed , D. Raha , A. M. Szekely , S. M. Weissman , M. Snyder , Genome Res. 2012, 22, 1668. 22955979 10.1101/gr.127761.111 PMC3431484

[149] J. M. Borin , R. Liu , Y. Wang , T. C. Wu , J. Chopyk , L. Huang , P. Kuo , C. Ghose , J. R. Meyer , X. M. Tu , B. Schnabl , D. T. Pride , Gut Microbes 2023, 15, 2236750. 37475473 10.1080/19490976.2023.2236750 PMC10364654

[150] J. Tomas , C. Mulet , A. Saffarian , J. B. Cavin , R. Ducroc , B. Regnault , C. Kun Tan , K. Duszka , R. Burcelin , W. Wahli , P. J. Sansonetti , T. Pédron , Proc Natl Acad Sci U S A 2016, 113, E5934. 27638207 10.1073/pnas.1612559113 PMC5056107

[151] H. C. Lin , JAMA, J. Am. Med. Assoc. 2004, 292, 852.

[152] M. Pimentel , E. J. Chow , H. C. Lin , Am. J. Gastroenterol. 2000, 95, 3503. 11151884 10.1111/j.1572-0241.2000.03368.x

[153] U. C. Ghoshal , R. Shukla , U. Ghoshal , Gut Liver 2017, 11, 196. 28274108 10.5009/gnl16126 PMC5347643

[154] E. C. Lauritano , M. Gabrielli , E. Scarpellini , A. Lupascu , M. Novi , S. Sottili , G. Vitale , V. Cesario , M. Serricchio , G. Cammarota , G. Gasbarrini , A. Gasbarrini , Am. J. Gastroenterol. 2008, 103, 2031. 18802998 10.1111/j.1572-0241.2008.02030.x

[155] Z. Wu , S. Huang , T. Li , N. Li , D. Han , B. Zhang , Z. Z. Xu , S. Zhang , J. Pang , S. Wang , G. Zhang , J. Zhao , J. Wang , Microbiome 2021, 9, 184. 34493333 10.1186/s40168-021-01115-9 PMC8424887

[156] S. M. Bahr , B. J. Weidemann , A. N. Castro , J. W. Walsh , O. Deleon , C. M. L. Burnett , N. A. Pearson , D. J. Murry , J. L. Grobe , J. R. Kirby , EBioMedicine 2015, 2, 1725. 26870798 10.1016/j.ebiom.2015.10.018 PMC4740326

[157] T. S. Rasmussen , C. M. J. Mentzel , M. R. Danielsen , R. R. Jakobsen , L. S. F. Zachariassen , J. L. Castro Mejia , A. Brunse , L. H. Hansen , C. H. F. Hansen , A. K. Hansen , D. S. Nielsen , Gut Microbes 2023, 15, 2208504. 37150906 10.1080/19490976.2023.2208504 PMC10167882

[158] L. Maier , C. V. Goemans , J. Wirbel , M. Kuhn , C. Eberl , M. Pruteanu , P. Müller , S. Garcia‐Santamarina , E. Cacace , B. Zhang , C. Gekeler , T. Banerjee , E. E. Anderson , A. Milanese , U. Löber , S. K. Forslund , K. R. Patil , M. Zimmermann , B. Stecher , G. Zeller , P. Bork , A. Typas , Nature 2021, 599, 120. 34646011 10.1038/s41586-021-03986-2 PMC7612847

[159] D. F. Niño , C. P. Sodhi , D. J. Hackam , Nat. Rev. Gastroenterol. Hepatol. 2016, 13, 590. 27534694 10.1038/nrgastro.2016.119 PMC5124124

[160] C. Chehoud , A. Dryga , Y. Hwang , D. Nagy‐Szakal , E. B. Hollister , R. A. Luna , J. Versalovic , R. Kellermayer , F. D. Bushman , mBio 2016, 7, 00322. 10.1128/mBio.00322-16 PMC4817255 27025251

[161] B. Barberio , D. Massimi , L. Bonfante , S. Facchin , L. Calò , M. Trevenzoli , E. V. Savarino , A. M. Cattelan , Therap Adv Gastroenterol 2020, 13, 175628482093458. 10.1177/1756284820934589 PMC7425245 32849912

[162] D. Rachmilewitz , F. Karmeli , K. Takabayashi , T. Hayashi , L. Leider‐Trejo , J. Lee , L. M. Leoni , E. Raz , Gastroenterology 2002, 122, 1428. 11984528 10.1053/gast.2002.32994

[163] D. Rachmilewitz , K. Katakura , F. Karmeli , T. Hayashi , C. Reinus , B. Rudensky , S. Akira , K. Takeda , J. Lee , K. Takabayashi , E. Raz , Gastroenterology 2004, 126, 520. 14762789 10.1053/j.gastro.2003.11.019

[164] H. G. Hampton , B. N. J. Watson , P. C. Fineran , Nature 2020, 577, 327. 31942051 10.1038/s41586-019-1894-8

[165] H. Raeisi , M. Noori , M. Azimirad , S. R. Mohebbi , H. Asadzadeh Aghdaei , A. Yadegar , M. R. Zali , Gut Pathog 2023, 15, 21. 37161478 10.1186/s13099-023-00550-3 PMC10169144

[166] W. Yan , P. Banerjee , M. Xu , S. Mukhopadhyay , M. Ip , N. B. Carrigy , D. Lechuga‐Ballesteros , K. K. W. To , S. S. Y. Leung , Adv Drug Deliv Rev 2021, 176, 113864. 34271022 10.1016/j.addr.2021.113864

[167] J. Heuler , L. C. Fortier , X. Sun , FEMS Microbiol. Rev. 2021, 45. 10.1093/femsre/fuab012 PMC8498794 33580957

[168] D. Wu , C. Zhang , Y. Liu , J. Yao , X. Yang , S. Wu , J. Du , X. Yang , J Microbiol Immunol Infect 2023. 10.1016/j.jmii.2023.02.005 36890066

[169] I. Mukhopadhya , J. P. Segal , S. R. Carding , A. L. Hart , G. L. Hold , Therap Adv Gastroenterol 2019, 12, 175628481983662. 10.1177/1756284819836620 PMC6435874 30936943

[170] M. Schwartz , M. Gluck , S. Koon , Am. J. Gastroenterol. 2013, 108, 1367. 10.1038/ajg.2013.164 23912408

[171] D. J. Lane , B. Pace , G. J. Olsen , D. A. Stahl , M. L. Sogin , N. R. Pace , Proc Natl Acad Sci U S A 1985, 82, 6955. 2413450 10.1073/pnas.82.20.6955 PMC391288

[172] J. Jovel , J. Patterson , W. Wang , N. Hotte , S. O'Keefe , T. Mitchel , T. Perry , D. Kao , A. L. Mason , K. L. Madsen , G. K. Wong , Front Microbiol 2016, 7, 459. 27148170 10.3389/fmicb.2016.00459 PMC4837688

[173] F. Cottier , K. G. Srinivasan , M. Yurieva , W. Liao , M. Poidinger , F. Zolezzi , N. Pavelka , NPJ Biofilms Microbiomes 2018, 4, 2. 29367879 10.1038/s41522-017-0046-x PMC5773663

[174] C. R. Wensel , J. L. Pluznick , S. L. Salzberg , C. L. Sears , J. Clin. Invest. 2022, 132, e154944. 35362479 10.1172/JCI154944 PMC8970668

[175] V. Aggarwala , I. Mogno , Z. Li , C. Yang , G. J. Britton , A. Chen‐Liaw , J. Mitcham , G. Bongers , D. Gevers , J. C. Clemente , J. F. Colombel , A. Grinspan , J. Faith , Nat. Microbiol. 2021, 6, 1309. 34580445 10.1038/s41564-021-00966-0 PMC8993687

[176] R. He , P. Li , J. Wang , B. Cui , F. Zhang , F. Zhao , Gut Microbes 2022, 14, 2100197. 35854629 10.1080/19490976.2022.2100197 PMC9302524

[177] D. Podlesny , C. Arze , E. Dorner , S. Verma , S. Dutta , J. Walter , W. F. Fricke , Microbiome 2022, 10, 53. 35337386 10.1186/s40168-022-01251-w PMC8951724

[178] X.‐B. Qian , T. Chen , Y.‐P. Xu , L. Chen , F.‐X. Sun , M. P. Lu , Y. X. Liu , Chin Med J (Engl) 2020, 133, 1844. 32604176 10.1097/CM9.0000000000000871 PMC7469990

[179] E. L. Van Dijk , Y. Jaszczyszyn , D. Naquin , C. Thermes , Trends Genet. 2018, 34, 666. 29941292 10.1016/j.tig.2018.05.008

[180] A. H. Laszlo , I. M. Derrington , B. C. Ross , H. Brinkerhoff , A. Adey , I. C. Nova , J. M. Craig , K. W. Langford , J. M. Samson , R. Daza , K. Doering , J. Shendure , J. H. Gundlach , Nat. Biotechnol. 2014, 32, 829. 24964173 10.1038/nbt.2950 PMC4126851

[181] J. Cao , Y. Zhang , M. Dai , J. Xu , L. Chen , F. Zhang , N. Zhao , J. Wang , Med Microecol 2020, 4, 100012.

[182] D. Deamer , M. Akeson , D. Branton , Nat. Biotechnol. 2016, 34, 518. 27153285 10.1038/nbt.3423 PMC6733523

[183] H. Jin , L. You , F. Zhao , S. Li , T. Ma , L.‐Y. Kwok , H. Xu , Z. Sun , Z. Sun , Gut Microbes 2022, 14, 2021790. 35067170 10.1080/19490976.2021.2021790 PMC8786330

[184] N. Zhao , J. Cao , J. Xu , B. Liu , B. Liu , D. Chen , B. Xia , L. Chen , W. Zhang , Y. Zhang , X. Zhang , Z. Duan , K. Wang , F. Xie , K. Xiao , W. Yan , L. Xie , H. Zhou , J. Wang , Adv. Sci. (Weinh) 2021, 8, e2102593. 34687159 10.1002/advs.202102593 PMC8655164

[185] F. Bäckhed , J. Roswall , Y. Peng , Q. Feng , H. Jia , P. Kovatcheva‐Datchary , Y. Li , Y. Xia , H. Xie , H. Zhong , M. T. Khan , J. Zhang , J. Li , L. Xiao , J. Al‐Aama , D. Zhang , Y. S. Lee , D. Kotowska , C. Colding , V. Tremaroli , Y. Yin , S. Bergman , X. Xu , L. Madsen , K. Kristiansen , J. Dahlgren , J. Wang , Cell Host Microbe 2015, 17, 852. 26308884 10.1016/j.chom.2015.05.012

[186] M. Zimmermann , M. Zimmermann‐Kogadeeva , R. Wegmann , A. L. Goodman , Nature 2019, 570, 462. 31158845 10.1038/s41586-019-1291-3 PMC6597290

[187] G. Cammarota , G. Ianiro , A. Ahern , C. Carbone , A. Temko , M. J. Claesson , A. Gasbarrini , G. Tortora , Nat. Rev. Gastroenterol. Hepatol. 2020, 17, 635. 32647386 10.1038/s41575-020-0327-3

[188] S. Khanna , M. Assi , C. Lee , D. Yoho , T. Louie , W. Knapple , H. Aguilar , J. Garcia‐Diaz , G. P. Wang , S. M. Berry , J. Marion , X. Su , T. Braun , L. Bancke , P. Feuerstadt , Drugs 2022, 82, 1539. 36342618 10.1007/s40265-022-01805-0 PMC9652262

[189] S. Ke , S. T. Weiss , Y.‐Y. Liu , Trends Mol Med 2022, 28, 619. 35781423 10.1016/j.molmed.2022.05.005 PMC9339459

[190] X. Bai , Y. Sun , Y. Li , M. Li , Z. Cao , Z. Huang , F. Zhang , P. Yan , L. Wang , J. Luo , J. Wu , D. Fan , H. Chen , M. Zhi , P. Lan , Z. Zeng , X. Wu , Y. Miao , T. Zuo , Microbiome 2022, 10, 147. 36100953 10.1186/s40168-022-01335-7 PMC9469561

[191] Y. Sun , T. Zuo , C. P. Cheung , W. Gu , Y. Wan , F. Zhang , N. Chen , H. Zhan , Y. K. Yeoh , J. Niu , Y. Du , F. Zhang , Y. Wen , J. Yu , J. J. Y. Sung , P. K. S. Chan , F. K. L. Chan , K. Wang , S. C. Ng , Y. Miao , Gastroenterology 2021, 160, 272. 32956679 10.1053/j.gastro.2020.09.014

[192] T. Zuo , Y. Sun , Y. Wan , Y. K. Yeoh , F. Zhang , C. P. Cheung , N. Chen , J. Luo , W. Wang , J. J. Y. Sung , P. K. S. Chan , K. Wang , F. K. L. Chan , Y. Miao , S. C. Ng , Cell Host Microbe 2020, 28, 741. 32910902 10.1016/j.chom.2020.08.005

